# Weight Change Between Pregnancies and Mortality Over 50 Years of Follow‐Up

**DOI:** 10.1002/oby.70190

**Published:** 2026-05-20

**Authors:** Yajnaseni Chakraborti, Sunni L. Mumford, Edwina H. Yeung, Katherine L. Grantz, Pauline Mendola, James L. Mills, Ellen C. Caniglia, Colleen M. Brensinger, Cuilin Zhang, Enrique F. Schisterman, Stefanie N. Hinkle

**Affiliations:** ^1^ Department of Biostatistics, Epidemiology and Informatics Perelman School of Medicine, University of Pennsylvania Philadelphia Pennsylvania USA; ^2^ Department of Obstetrics and Gynecology Perelman School of Medicine, University of Pennsylvania Philadelphia Pennsylvania USA; ^3^ Epidemiology Branch, Division of Population Health Research, Division of Intramural Research Eunice Kennedy Shriver National Institute of Child Health and Human Development, National Institutes of Health Bethesda Maryland USA; ^4^ Department of Epidemiology and Environmental Health School of Public Health and Health Professions, University of Buffalo Buffalo New York USA; ^5^ Biostatistics Analysis Center, Center for Clinical Epidemiology and Biostatistics, Perelman School of Medicine, University of Pennsylvania Philadelphia Pennsylvania USA; ^6^ Department of Nutrition Harvard T.H. Chan School of Public Health Boston Massachusetts USA; ^7^ Global Centre for Asian Women's Health, Yong Loo Lin School of Medicine, National University of Singapore Singapore; ^8^ Department of Obstetrics and Gynecology Yong Loo Lin School of Medicine, National University of Singapore Singapore; ^9^ Bia‐Echo Asia Centre for Reproductive Longevity & Equality, Yong Loo Lin School of Medicine, National University of Singapore Singapore

**Keywords:** mortality, pregnancy, weight change

## Abstract

**Objective:**

This study aimed to evaluate the link between postpartum weight retention (PPWR) and long‐term mortality.

**Methods:**

In this secondary analysis of 8165 women with ≥ 2 pregnancies in the Collaborative Perinatal Project (CPP), U.S., 1959–1966, with vital status follow‐up through 2016, we used interconception weight change (ICWC) and interpregnancy weight change (IPWC) as proxies of PPWR. ICWC was defined as the difference between self‐reported pre‐pregnancy weight from the 1st and 2nd CPP pregnancies, while IPWC was the difference between weight recorded at delivery of the 1st and pre‐pregnancy weight of the 2nd CPP pregnancies. All‐cause and cause‐specific mortality models were adjusted for sociodemographic, behavioral, clinical, and pregnancy‐related characteristics from the 1st CPP pregnancy.

**Results:**

Compared to women with ICWC > 0 to 1.8 kg (quintile 3), those with ICWC > −1.4 to 0 kg (quintile 2) had a lower risk of all‐cause mortality (aHR [95% CI]: 0.85 [0.76–0.96]), with the most notable reduction observed in diabetes‐related risk of mortality (aHR [95% CI]: 0.45 [0.23–0.87]). Lower quintiles of IPWC (i.e., greater weight loss) were suggestive of reduced all‐cause and cause‐specific risk of mortality, though the estimates were not statistically significant.

**Conclusions:**

Minimizing PPWR was linked to reduced mortality risk over 50 years of follow‐up.

## Study Importance

- What is already known?
  ○ The prevalence of overweight and obesity is steadily rising among women of reproductive age, with the risk of obesity being twice as high among those who have given birth compared to those who have not.
  ○ Only about one in four women return to their pre‐pregnancy weight, with postpartum weight retention (PPWR) being associated with adverse cardiometabolic parameters that emerge within the first year after childbirth and persist over the long term.
- What does this study add?
  ○ Women who returned to or maintained a weight slightly below their pre‐pregnancy weight had lower risk of all‐cause mortality and diabetes‐related mortality compared to those with moderate PPWR between pregnancies, even after adjusting for gestational weight gain.
  ○ Women with low risk at baseline, i.e., a normal pre‐pregnancy BMI, may still face increased risk of earlier mortality if they retain or gain weight in the postpartum period.
- How might these results change the direction of research or the focus of clinical practice?
  ○ These results underscore the benefit of postpartum weight loss and highlight the need for effective strategies to prevent PPWR or new weight gain in all women, as the associated risks persist even after adjusting for baseline BMI and gestational weight gain.

## 1 Introduction

Over the past three decades, the prevalence of overweight and obesity among women of reproductive age has steadily increased [1, 2, 3]. Currently, more than one in four women between the ages of 20 and 39 in the U.S. are living with overweight (27.5%) or obesity (36.8%) [1, 2, 4]. For many women, weight gained during pregnancy may contribute to the development of overweight or obesity [5]. In fact, the 5‐year incidence of obesity is twice as high among women who have had a child compared to those who have never given birth [6]. A comparable pattern of risk was reported in a separate study investigating childbearing and weight gain over 10 years [7]. A probable mechanism underlying this risk of obesity associated with parity is postpartum weight retention (PPWR), which refers to the weight a person retains after giving birth, in addition to the weight gained during pregnancy.

Only about one in four women return to their pre‐pregnancy weight by 1 year post partum [8]. For instance, a U.S.‐based prospective cohort study by Endres et al. [8], found that approximately 75% of participants weighed more 1 year after giving birth than they did before pregnancy—at 1 year post partum, 47.4% retained more than 10 lb and 24.2% retained more than 20 lb. Further, among those with a normal pre‐pregnancy body mass index (BMI), 33% were living with overweight or obesity at 1 year post partum [8]. Legro et al. [9] reported similar findings in their study of first‐time mothers in Pennsylvania, where 23.7% retained between 1 and 9 lb, and 23.9% retained 10 or more lb at 1 year post partum. Among those with a normal pre‐pregnancy BMI, 12% were living with overweight or obesity at 1 year post partum [9].

Indeed, gestational weight gain (GWG) is a likely contributor to PPWR [5, 10, 11, 12], with studies showing associations between excessive GWG and greater overall and abdominal adiposity even 8–15 years post partum [13]. However, GWG is not the only factor influencing PPWR; other contributors, such as lifestyle behaviors and breastfeeding, may also affect whether weight is retained post partum [12, 14, 15, 16, 17, 18, 19, 20, 21]. Given that multiple factors contribute to PPWR, it is important to examine its impact on health as a distinct exposure, rather than viewing it solely as a consequence of GWG [10]. In fact, one study found that postpartum weight trajectories after pregnancy were associated with excess weight and increased adiposity risk at 3 years post partum, even after adjusting for GWG [12].

Additionally, the implications of PPWR on cardiometabolic health are well documented with adverse cardiometabolic profiles often emerging within the first year post partum [22], and elevated risks of prediabetes or diabetes [23] and poor cardiometabolic profiles [12, 23], as well as hypertensive and cardiovascular disorders [24], continuing to manifest at 3 years [12, 23], 5 years [23], and 16 years [24] post partum.

Given the evidence of sustained alterations in weight and cardiometabolic parameters, it is reasonable to hypothesize that PPWR may be related to long‐term mortality, potentially independent of pre‐pregnancy BMI and GWG; yet this remains unexplored. To evaluate this hypothesis, we estimated the risk of all‐cause mortality over 50 years of follow‐up post pregnancy, in relation to two analytic exposures: (1) interconception weight change (ICWC) and (2) interpregnancy weight change (IPWC), which are operational proxies for PPWR capturing maternal weight dynamics across distinct reproductive intervals. Additionally, given the existing evidence linking PPWR to cardiovascular and metabolic disorders [25, 26], we also examined the cardiovascular, diabetes, and kidney‐specific mortality risks associated with these two exposures.

## 2 Methods

This study is a secondary analysis of data collected from the Collaborative Perinatal Project (CPP) and the CPP Mortality Linkage Study. The CPP was a multicenter observational prospective cohort study conducted in the U.S. between 1959 and 1966 to assess the effects of pregnancy and perinatal complications on birth and child outcomes [27, 28, 29, 30, 31, 32]. A total of 48,917 pregnant women were enrolled at their first prenatal visit and were followed across multiple pregnancies, resulting in 58,760 registered pregnancies. The mortality status of this cohort as of December 31, 2016, was later determined through the CPP Mortality Linkage Study [33] to explore the long‐term implications of gestational exposures. This analysis utilizes data from the first two pregnancies for participants with two or more CPP pregnancies (*n* = 8165). It is important to note that the 1st pregnancy registered within CPP was not necessarily an individual's 1st pregnancy overall. For the purposes of this study, we have used the term “1st pregnancy” to refer specifically to the 1st pregnancy recorded in CPP.

There were no specific regulations for approving research involving human subjects at the time CPP began in the late 1950s, yet general informed consent for participation was obtained. IRB approval for the CPP Mortality Linkage Study was obtained by the Eunice Kennedy Shriver National Institute of Child Health and Human Development (IRB00011862, approved in 2015) and the Emmes Corporation (IRB00000879, approved in 2013)—the two organizations responsible for abstracting identifying information from historic CPP records and facilitating the data linkages to the National Death Index (NDI) and the Social Security Death Master File (SSDMF). Mortality data were linked through December 31, 2016. The University of Pennsylvania IRB entered into a reliance agreement with the NICHD IRB for oversight of this study.

### 2.1 Exposures

**FIGURE 1:**
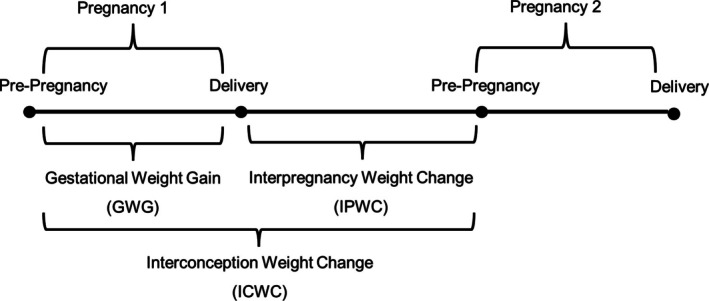
Exposures and timeline of measurement.

Participants self‐reported their pre‐pregnancy weight at their first prenatal visit for each CPP pregnancy. Participants' weight at each prenatal visit and delivery admission was abstracted from medical records. This analysis conceptualized PPWR using two different weight exposures based on the participants' consecutive 1st and 2nd CPP pregnancies (Figure 1). The primary exposure was ICWC, which represented the overall weight change between the 1st and 2nd CPP pregnancies. Specifically, ICWC was calculated as the difference between pre‐pregnancy weight of the 2nd and 1st pregnancies. We also examined a secondary exposure, IPWC, which focused solely on weight change in the interpregnancy interval (IPI). Specifically, IPWC was calculated as the difference between pre‐pregnancy weight of the 2nd CPP pregnancy and weight recorded at admission for delivery of the 1st CPP pregnancy. We preprocessed height and weight measurements by identifying and setting implausible values to missing based on predefined thresholds (detailed specifications in Appendix A in online Supporting Information).

### 2.2 Outcomes

The primary outcome of interest was time to all‐cause mortality, defined as the time from the start of follow‐up to the date of death or December 31, 2016, whichever occurred first. Follow‐up started at the date of the last menstrual period (LMP) of the participant's 2nd CPP pregnancy. All‐cause mortality was ascertained through the NDI; deaths not identified in the NDI were captured using the SSDMF. The NDI includes all U.S. death records from 1979 onward, with matches identified through probabilistic linkage. Although the SSDMF includes deaths dating back to 1958, the NDI is considered a more accurate and comprehensive source of mortality and also includes cause of death [33, 34]. Overall, 89% of the cohort's death information was sourced from the NDI. Given the link between PPWR and cardiometabolic health [12, 23], secondary outcomes considered in this study were time to cause‐specific mortality related to cardiovascular disease (I00‐I78, I80‐I99), diabetes (E10‐E14), and kidney conditions (N00‐N07, N17‐N19, N25‐N27) where the underlying cause of death was coded using ICD‐10 (International Classification of Diseases, Tenth Revision) and comparable ICD‐9 codes. Some misclassification of cause of death is possible. Because only approximately 3% of deaths occurred before 1979 [33], the impact of missing cause‐of‐death information is expected to be minimal.

### 2.3 Covariates

Covariates were reported by participants during the first prenatal visit of their 1st CPP pregnancy and included information on age, frequency of smoking, race, marital status, income, education, number of prior pregnancies, hypertensive disorders of pregnancy [35], diabetes prior to pregnancy, prior cardiovascular diseases, prior respiratory diseases, prior renal disease, prior neurological conditions, prior cancer/tumor status, study site, pre‐pregnancy weight, and height. Race was conceptualized as a social determinant of health in this study. BMI (kg/m^2^) was calculated using self‐reported height and pre‐pregnancy weight. Additional 1st CPP pregnancy variables of interest included in this study were pregnancy plurality, gestational age at delivery, pregnancy outcomes, GWG, and the interconception and interpregnancy intervals. Details on how these variables were derived and incorporated at different analytical steps of the study are provided in Table S1.

### 2.4 Statistical Analysis

Post data cleaning and processing, the participant characteristics were summarized for each quintile of ICWC. Missing or implausible exposure, covariate, and outcome data were imputed via multiple imputation using chained equations. Under the missing at random assumption, 10 imputed datasets were generated for estimating pooled hazard ratios (HR) and the respective 95% confidence interval (CI). Multiple imputation was conducted using the R package *mice* [36]. All other data cleaning, processing, and analyses were conducted using SAS (SAS Institute Inc.).

The study sample was a subset of the CPP cohort with at least two pregnancies in CPP. Thus, to overcome the potential bias due to this inherent selection process and to ensure that the pooled hazard ratio estimates are generalizable to the entire CPP target population, inverse probability weights (IPW) were used to reweight the outcomes data. The IPW were calculated as the ratio of two probabilities: the marginal probability of having a 2nd CPP pregnancy as the numerator and the conditional probability of a 2nd CPP pregnancy as the denominator, where the conditional probability model included a wide range of variables as described in Table S1. The Kaplan–Meier and the log[−log S(t)] plots were used to visually assess proportionality, followed by Schoenfeld residual analyses to confirm that the proportional hazards assumption was satisfied for both exposures.

### 2.5 Analyses With Primary Exposure ICWC

ICWC was categorized into quintiles to identify any potential nonlinear associations between the exposure and the outcome. The risk of all‐cause mortality associated with quintiles of ICWC was estimated using IPW weighted Cox proportional hazard models. Three covariate adjustment scenarios were applied: (1) unadjusted, (2) adjusted for confounders, including sociodemographic characteristics, smoking status, reproductive and medical history, and study site; and (3) additionally adjusted for interconception interval (ICI), i.e., the time difference between the LMP of the 1st and 2nd CPP pregnancies, to assess if time between two pregnancies contributed to weight change.

Multiple sensitivity analyses were conducted. First, to elucidate the effect of extreme weight loss or weight gain, we separated the top and bottom 1% of ICWC values following an initial review of the data distribution, thus rendering seven different categories of ICWC. Second, we conducted a stratified analysis wherein the quintiles of ICWC were reassessed within BMI categories: < 18.5, 18.5–24.9, 25.0–29.9, ≥ 30.0. However, given the small sample sizes within some of the BMI category‐specific quintiles, the risk of all‐cause mortality was estimated only for those with normal (18.5–24.9) pre‐pregnancy BMI (70.5%, *n* = 5754).

Cause‐specific mortality risks due to cardiovascular, diabetes, and kidney conditions associated with quintiles of ICWC were estimated using cause‐specific hazard models, which model the instantaneous hazard of death from a specific cause among individuals who remain event‐free, assuming independent censoring by competing causes conditional on covariates [37]. Cause‐specific mortality risks associated with the BMI‐specific quintiles were not implemented due to small sizes. Details of the model specifications are provided in Table S1.

### 2.6 Analyses With Secondary Exposure IPWC

IPWC was also categorized into quintiles. The risk of all‐cause mortality associated with quintiles of IPWC and BMI‐specific quintiles of IPWC was estimated using similar modeling approaches as the ICWC analyses. The adjusted models additionally accounted for 1st pregnancy GWG and model 3 alternatively adjusted for the IPI, i.e., the time difference between the 1st pregnancy delivery date and the LMP of the 2nd pregnancy.

We completed multiple sensitivity analyses on IPWC. Given that the majority of the study participants lost weight and only a small fraction had weight gain during the IPI, a third scenario distinguishing weight gain as a separate category from the fifth quintile of IPWC was also evaluated to tease out the risk associated with postpartum weight gain. This third scenario was also implemented to models with BMI‐specific quintiles of IPWC. In addition, we repeated the all‐cause mortality analysis with IPWC quintiles, and with the weight gain group separated out from the fifth quintile, to include additional covariate adjustment and account for three potential confounding mechanisms. First, we adjusted for birth weight as a covariate given that our derivation of IPWC incorporates the weight of the baby and therefore may account partially for the weight change patterns observed during the IPI in this study. Secondly, there might have been an impact of shorter gestation among women delivering pre‐term on IPWC measures as well as on mortality—women delivering pre‐term were likely to have less GWG, and pre‐term delivery is known to be associated with increased mortality by cardiovascular disease [38]. Therefore, we also ran a separate model including gestational age at delivery as a covariate. Finally, although pregnancy outcome is presumed to influence IPWC predominantly via postpartum experiences and behaviors, a direct pathway cannot be completely dismissed. Thus, we fitted a third model that included pregnancy outcome as an additional covariate.

## 3 Results

For both ICWC and IPWC, Kaplan–Meier and log[−log S(t)] plots (Figure S1) showed parallel curves across quintiles, and Schoenfeld residuals (Figure S2) showed no systematic deviation from zero over time, i.e., no evidence of heterogeneity over follow‐up, indicating no violation of the proportional hazards assumption.

### 3.1 Risk of Mortality Associated With ICWC

The majority of women (~56%, *n* = 4141) had a higher weight at the beginning of their 2nd pregnancy than their 1st pregnancy (Table 1), and the median ICWC was 0.91 kg (interquartile range [IQR]: −0.45, 3.63) (Figure S3). The distributions of most participant characteristics were similar across quintiles. Some notable exceptions include the increase in the percentage of nonsmokers as ICWC increased, with the highest percentage of nonsmokers in the highest quintile of ICWC. The interval between conceptions for most quintiles of ICWC (first, third, and fourth quintiles) averaged around 2 years (~24 months) but was slightly shorter for the second quintile (~22 months) and longer (~27 months) for the fifth quintile of ICWC. The median follow‐up for this study sample was 54 years (IQR: 47, 56), and 41.7% (*n* = 3405) died during the study period (Table 2).

**TABLE 1:** Summary of participant characteristics across quintiles of interconception weight change (ICWC).

Participant characteristics at 1st CPP pregnancy	ICWC quintile					
	Q1: ≤ −1.4 kg (*n* = 1501)	Q2: > −1.4 to 0 kg (*n* = 1780)	Q3: > 0 to 1.8 kg (*n* = 1269)	Q4: > 1.8 to 4.5 kg (*n* = 1621)	Q5: > 4.5 kg (*n* = 1251)	Missing (*n* = 743)
	*n* (%)	*n* (%)	*n* (%)	*n* (%)	*n* (%)	*n* (%)
Age (years)						
Mean (SD)	22.6 (5.3)	22.8 (5.3)	23.3 (5.5)	23.1 (5.5)	22.4 (5.1)	23.8 (5.7)
Missing	—	—	—	—	—	—
Race and ethnicity						
Black	690 (46.0)	811 (45.6)	549 (43.3)	738 (45.5)	623 (49.8)	321 (43.2)
Asian/other	18 (1.2)	18 (1.0)	12 (0.9)	8 (0.5)	5 (0.4)	8 (1.1)
Puerto Rican	43 (2.9)	63 (3.5)	23 (1.8)	65 (4.0)	41 (3.3)	57 (7.7)
White	750 (50.0)	888 (49.9)	685 (54.0)	810 (50.0)	582 (46.5)	357 (48.0)
Missing	—	—	—	—	—	—
Number of prior pregnancies						
0	615 (41.0)	650 (36.5)	485 (38.2)	612 (37.8)	520 (41.6)	244 (32.8)
1	280 (18.7)	382 (21.5)	252 (19.9)	337 (20.8)	222 (17.7)	126 (17.0)
2	184 (12.3)	266 (14.9)	187 (14.7)	211 (13.0)	157 (12.5)	87 (11.7)
3	155 (10.3)	153 (8.6)	118 (9.3)	157 (9.7)	114 (9.1)	96 (12.9)
4	261 (17.4)	315 (17.7)	216 (17.0)	297 (18.3)	229 (18.3)	154 (20.7)
Missing	6 (0.4)	14 (0.8)	11 (0.9)	7 (0.4)	9 (0.7)	36 (4.8)
Marital status						
Married/common law	1156 (77.0)	1406 (79.0)	1044 (82.3)	1295 (79.9)	992 (79.3)	597 (80.3)
Single	264 (17.6)	271 (15.2)	166 (13.1)	236 (14.6)	181 (14.5)	104 (14.0)
Widowed/divorced/separated	81 (5.4)	103 (5.8)	59 (4.6)	90 (5.6)	78 (6.2)	42 (5.7)
Missing	—	—	—	—	—	—
Smoking status						
Nonsmoker	697 (46.4)	859 (48.3)	654 (51.5)	883 (54.5)	736 (58.8)	354 (47.6)
< 1 pack per day	548 (36.5)	655 (36.8)	431 (34.0)	512 (31.6)	371 (29.7)	244 (32.8)
≥ 1 pack per day	241 (16.1)	247 (13.9)	168 (13.2)	210 (13.0)	133 (10.6)	103 (13.9)
Missing	15 (1.0)	19 (1.1)	16 (1.3)	16 (1.0)	11 (0.9)	42 (5.7)
Annual income						
≤ $1999	234 (15.6)	256 (14.4)	139 (11.0)	241 (14.9)	193 (15.4)	93 (12.5)
$2000–$3999	613 (40.8)	675 (37.9)	457 (36.0)	614 (37.9)	472 (37.7)	272 (36.6)
$4000–$5999	284 (18.9)	389 (21.9)	279 (22.0)	337 (20.8)	236 (18.9)	137 (18.4)
$6000–$7999	113 (7.5)	144 (8.1)	154 (12.1)	151 (9.3)	127 (10.2)	65 (8.7)
$8000–$9999	47 (3.1)	54 (3.0)	50 (3.9)	70 (4.3)	40 (3.2)	17 (2.3)
≥ $10,000	20 (1.3)	37 (2.1)	26 (2.0)	29 (1.8)	23 (1.8)	13 (1.7)
Missing	234 (15.6)	256 (14.4)	139 (11.0)	241 (14.9)	193 (15.4)	93 (12.5)
Education						
< High school	251 (16.7)	268 (15.1)	144 (11.3)	235 (14.5)	223 (17.8)	154 (20.7)
Some high school	622 (41.4)	765 (43.0)	480 (37.8)	664 (41.0)	509 (40.7)	259 (34.9)
High school graduate	421 (28.0)	504 (28.3)	435 (34.3)	522 (32.2)	391 (31.3)	178 (24.0)
≥ Some college	148 (9.9)	181 (10.2)	155 (12.2)	143 (8.8)	89 (7.1)	84 (11.3)
Missing	59 (3.9)	62 (3.5)	55 (4.3)	57 (3.5)	39 (3.1)	68 (9.2)
Hypertensive disorders of pregnancy						
Normotensive	1395 (92.9)	1692 (95.1)	1215 (95.7)	1505 (92.8)	1145 (91.5)	682 (91.8)
Chronic hypertension	33 (2.2)	37 (2.1)	21 (1.7)	38 (2.3)	50 (4.0)	25 (3.4)
Gestational hypertension	33 (2.2)	27 (1.5)	22 (1.7)	39 (2.4)	32 (2.6)	12 (1.6)
Preeclampsia/eclampsia	17 (1.1)	11 (0.6)	6 (0.5)	20 (1.2)	11 (0.9)	9 (1.2)
Superimposed preeclampsia/eclampsia	23 (1.5)	13 (0.7)	4 (0.3)	19 (1.2)	13 (1.0)	7 (0.9)
Missing	—	—	1 (0.1)	—	—	8 (1.1)
Prior diabetes^†^						
No	1472 (98.1)	1755 (98.6)	1245 (98.1)	1599 (98.6)	1229 (98.2)	700 (94.2)
Yes	29 (1.9)	25 (1.4)	24 (1.9)	22 (1.4)	22 (1.8)	36 (4.8)
Missing	—	—	—	—	—	7 (0.9)
Prior cardiovascular conditions^‡^						
No	1294 (86.2)	1561 (87.7)	1121 (88.3)	1420 (87.6)	1087 (86.9)	653 (87.9)
Yes	207 (13.8)	218 (12.2)	147 (11.6)	201 (12.4)	164 (13.1)	77 (10.4)
Missing	—	1 (0.1)	1 (0.1)	—	—	13 (1.7)
Prior respiratory conditions^§^						
No	1390 (92.6)	1636 (91.9)	1183 (93.2)	1503 (92.7)	1171 (93.6)	677 (91.1)
Yes	111 (7.4)	143 (8.0)	85 (6.7)	118 (7.3)	80 (6.4)	53 (7.1)
Missing	—	1 (0.1)	1 (0.1)	—	—	13 (1.7)
Prior renal conditions^¶^						
No	1438 (95.8)	1714 (96.3)	1213 (95.6)	1553 (95.8)	1206 (96.4)	689 (92.7)
Yes	63 (4.2)	65 (3.7)	55 (4.3)	68 (4.2)	45 (3.6)	41 (5.5)
Missing	—	1 (0.1)	1 (0.1)	—	—	13 (1.7)
Prior neurological conditions^||^						
No	1385 (92.3)	1646 (92.5)	1165 (91.8)	1508 (93.0)	1151 (92.0)	663 (89.2)
Yes	116 (7.7)	133 (7.5)	103 (8.1)	113 (7.0)	100 (8.0)	67 (9.0)
Missing	—	1 (0.1)	1 (0.1)	—	—	13 (1.7)
Prior cancer or tumors **						
No	1447 (96.4)	1732 (97.3)	1216 (95.8)	1576 (97.2)	1213 (97.0)	708 (95.3)
Yes	54 (3.6)	47 (2.6)	52 (4.1)	45 (2.8)	38 (3.0)	22 (3.0)
Missing	—	1 (0.1)	1 (0.1)	—	—	13 (1.7)
Pregnancy plurality						
Singleton	1487 (99.1)	1766 (99.2)	1254 (98.8)	1605 (99.0)	1245 (99.5)	703 (94.6)
Multiples	12 (0.8)	13 (0.7)	13 (1.0)	14 (0.9)	6 (0.5)	4 (0.5)
Missing	2 (0.1)	1 (0.1)	2 (0.2)	2 (0.1)	0 (0.0)	36 (4.8)
Study site						
Boston	408 (27.2)	474 (26.6)	444 (35.0)	536 (33.1)	363 (29.0)	248 (33.4)
Buffalo	61 (4.1)	99 (5.6)	64 (5.0)	64 (3.9)	31 (2.5)	22 (3.0)
New Orleans	29 (1.9)	31 (1.7)	16 (1.3)	31 (1.9)	30 (2.4)	4 (0.5)
New York/Columbia	11 (0.7)	13 (0.7)	11 (0.9)	20 (1.2)	10 (0.8)	5 (0.7)
Baltimore	111 (7.4)	118 (6.6)	97 (7.6)	110 (6.8)	94 (7.5)	61 (8.2)
Virginia	98 (6.5)	123 (6.9)	71 (5.6)	81 (5.0)	70 (5.6)	32 (4.3)
Minnesota	70 (4.7)	115 (6.5)	69 (5.4)	65 (4.0)	43 (3.4)	25 (3.4)
New York/medical college	50 (3.3)	67 (3.8)	19 (1.5)	52 (3.2)	42 (3.4)	46 (6.2)
Oregon	141 (9.4)	134 (7.5)	51 (4.0)	104 (6.4)	90 (7.2)	27 (3.6)
Pennsylvania	352 (23.5)	411 (23.1)	284 (22.4)	368 (22.7)	324 (25.9)	228 (30.7)
Providence	133 (8.9)	143 (8.0)	103 (8.1)	132 (8.1)	106 (8.5)	42 (5.7)
Tennessee	37 (2.5)	52 (2.9)	40 (3.2)	58 (3.6)	48 (3.8)	3 (0.4)
Missing	—	—	—	—	—	—
Height (cm)						
Mean (SD)	161.5 (6.2)	160.8 (6.3)	160.7 (6.2)	160.8 (6.3)	161.3 (6.4)	160.1 (7.1)
Missing	20 (1.3)	17 (1.0)	8 (0.6)	19 (1.2)	8 (0.6)	68 (9.2)
Pre‐pregnancy weight (kg)						
Mean (SD)	62.1 (12.1)	56.8 (9.9)	55.8 (9.2)	57.1 (9.9)	60.5 (11.8)	59.2 (11.7)
Missing	—	—	—	—	—	131 (17.6)
Pre‐pregnancy BMI (kg/m^2^)						
Mean (SD)	23.8 (4.4)	22.0 (3.6)	21.6 (3.4)	22.1 (3.5)	23.2 (4.2)	23.1 (4.3)
Median (IQR)	22.8 (20.7–25.8)	21.2 (19.5–23.4)	21.0 (19.5–23.0)	21.5 (19.7–23.6)	22.3 (20.2–25.3)	22.2 (20.1–24.8)
Missing	20 (1.3)	17 (1.0)	8 (0.6)	19 (1.2)	8 (0.6)	169 (22.7)
Pre‐pregnancy BMI (kg/m2) categories						
Underweight: < 18.5 kg/m^2^	63 (4.2)	189 (10.6)	163 (12.8)	175 (10.8)	88 (7.0)	52 (7.0)
Normal weight: 18.5–24.9 kg/m^2^	966 (64.4)	1302 (73.1)	940 (74.1)	1166 (71.9)	818 (65.4)	387 (52.1)
Overweight: 25.0–29.9 kg/m^2^	315 (21.0)	210 (11.8)	121 (9.5)	205 (12.6)	237 (18.9)	90 (12.1)
Obesity: > 30 kg/m^2^	137 (9.1)	62 (3.5)	37 (2.9)	56 (3.5)	100 (8.0)	45 (6.1)
Missing	20 (1.3)	17 (1.0)	8 (0.6)	19 (1.2)	8 (0.6)	169 (22.7)
Interconception interval (days) *						
Mean (SD)	710.6 (352.3)	662.3 (330.6)	693.6 (356.3)	703.0 (372.5)	793.3 (426.3)	725.4 (398.1)
Missing	5 (0.3)	2 (0.1)	—	1 (0.1)	—	655 (88.2)

*Note*: ^†^Type unknown. ^‡^Cardiovascular conditions included hypertension, rheumatic fever, and any other cardiovascular diseases. ^§^Respiratory conditions included tuberculosis, asthma, other chronic pulmonary diseases, and other conditions requiring thoracic surgery. ^¶^Renal conditions included pyelitis, glomerulonephritis, and other conditions requiring kidney, urinary, or bladder surgery. ^||^Neurological conditions included neuromuscular diseases, convulsive disorders psychosis, alcohol or drug addiction, or other neurological diseases. **Cancer and tumors included any history of cancer and gastrointestinal, kidney, urinary, bladder, or gynecological tumors. *Interconception Interval calculated as the time difference between the last menstrual period of the 1st and 2nd CPP pregnancies.

**TABLE 2:** Distribution of outcome of interest across quintiles of interconception weight change (ICWC).

Outcomes of interest	ICWC quintile					
	Q1: ≤ −1.4 kg (*n* = 1501)	Q2: > −1.4 to 0 kg (*n* = 1780)	Q3: > 0 to 1.8 kg (*n* = 1269)	Q4: > 1.8 to 4.5 kg (*n* = 1621)	Q5: > 4.5 kg (*n* = 1251)	Missing (*n* = 743)
	*n* (%)	*n* (%)	*n* (%)	*n* (%)	*n* (%)	*n* (%)
Vital status						
Alive	865 (57.6)	1067 (59.9)	752 (59.3)	941 (58.1)	712 (56.9)	423 (56.9)
Dead	636 (42.4)	713 (40.1)	517 (40.7)	680 (41.9)	539 (43.1)	320 (43.1)
Missing	—	—	—	—	—	—
Cause of death						
Cardiovascular	162 (25.5)	196 (27.5)	119 (23.0)	184 (27.1)	149 (27.6)	89 (27.8)
Diabetes	28 (4.4)	19 (2.7)	22 (4.3)	22 (3.2)	27 (5.0)	18 (5.6)
Kidney	8 (1.3)	8 (1.1)	10 (1.9)	14 (2.1)	14 (2.6)	5 (1.6)
Missing	61 (4.1)	68 (3.8)	46 (3.6)	64 (3.9)	56 (4.5)	30 (4.0)

**FIGURE 2:**
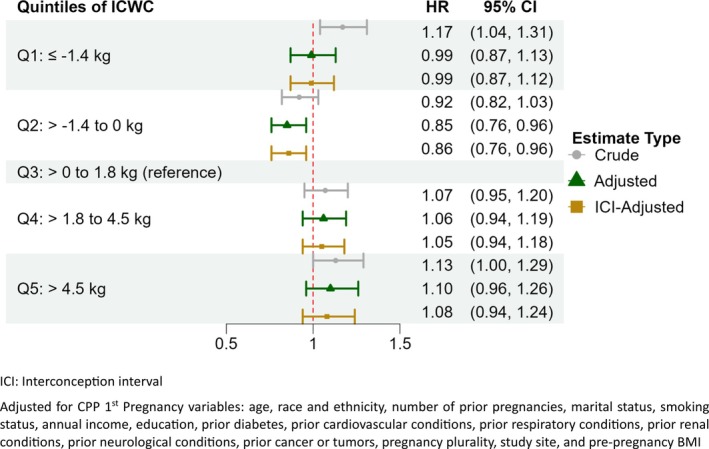
Risk of all‐cause mortality associated with quintiles of interconception weight change (ICWC). [Color figure can be viewed at wileyonlinelibrary.com]

Results from the adjusted model (Figure 2) demonstrated that compared to ICWC > 0 kg to 1.8 kg (Q3), ICWC > −1.4 to 0 kg (Q2) was associated with lower all‐cause mortality (aHR [95% CI]: 0.85 [0.76–0.96]). Adjusting for ICI did not yield any meaningful changes in the estimates.

Distinguishing the top and bottom 1% of ICWC (Figure S4) showed that very high ICWC > 17.2 kg (aHR [95% CI]: 1.57 [1.10–2.23]) and very low ICWC ≤ −10.4 kg (aHR [95% CI]: 1.15 [0.83–1.59]) were associated with higher all‐cause mortality compared to Q3 of ICWC; however, the results lacked precision.

Among those with normal pre‐pregnancy BMI at their 1st CPP pregnancy, ICWC > −0.9 to 0 kg was associated with a reduced risk of all‐cause mortality (aHR [95% CI]: 0.85 [0.73–0.99]) compared to ICWC > 0 to 1.8 kg (Table S2).

Similar patterns of cause‐specific mortality risks among women in Q2 and Q5 of ICWC were observed, although the estimates were imprecise due to limited sample sizes (Table S3), with the notable exception of ICWC > −1.4 to 0 kg (Q2) which was associated with a lower risk of mortality due to diabetes (aHR [95% CI]: 0.45 [0.23–0.87]).

### 3.2 Risk of Mortality Associated With IPWC

**FIGURE 3:**
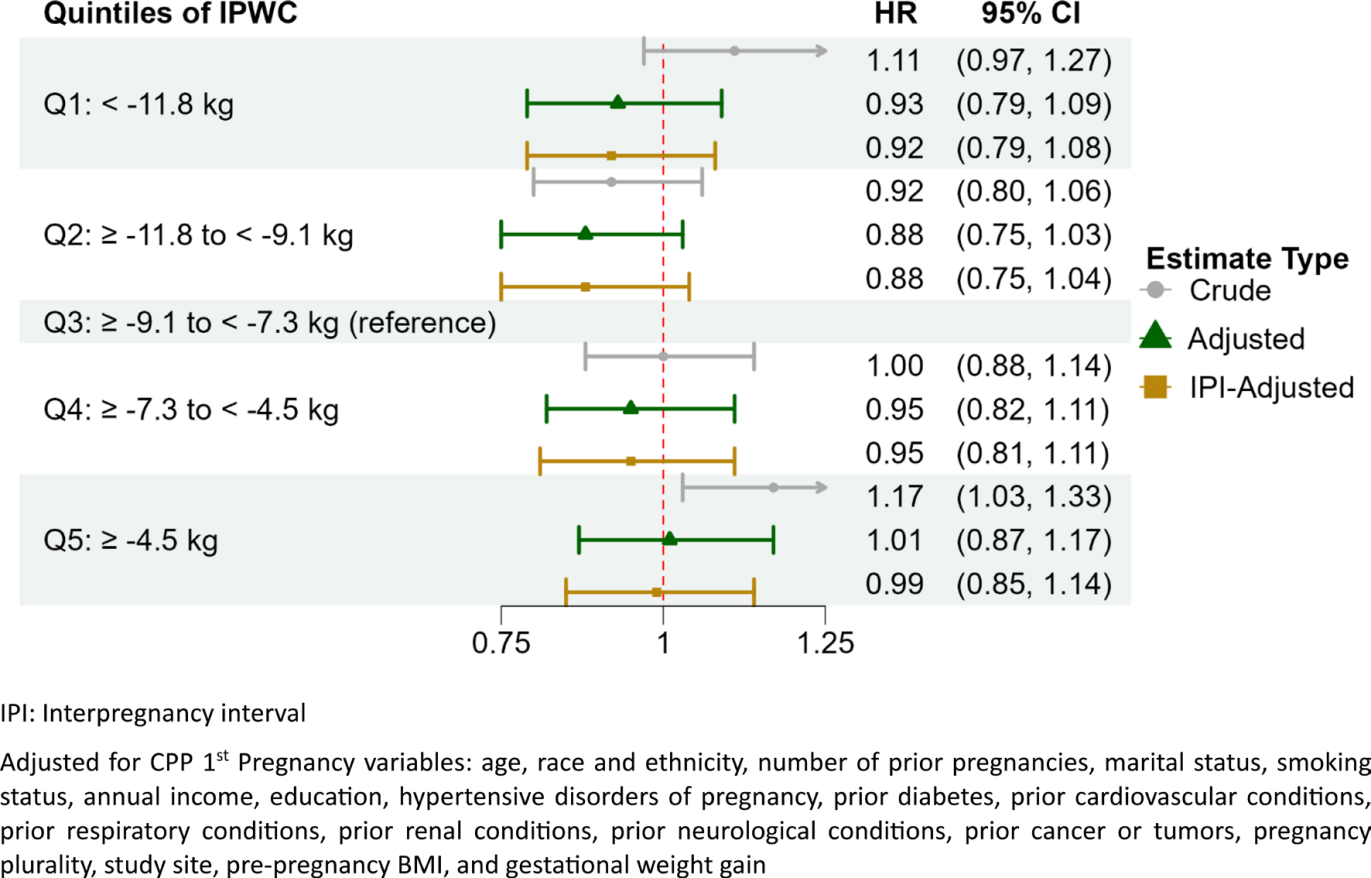
Risk of all‐cause mortality associated with quintiles of interpregnancy weight change (IPWC). [Color figure can be viewed at wileyonlinelibrary.com]

Approximately 94% of participants experienced weight loss during the postpartum period (median IPWC: −8.16 kg [IQR:−4.9, −11.34]) (Figure S3). Table S4 describes the participant characteristics according to IPWC. GWG was highest among those in Q1 (mean 13.8 kg [SD 4.5]) and lowest among those in Q5 (6.3 kg [4.8]). The IPI increased slightly from 1.2 years among those in Q1 to 1.4 years among those in Q5. We found no significant associations between IPWC and all‐cause mortality (Figure 3). Nevertheless, reduced risk of all‐cause mortality was suggested among those in Q1, Q2, and Q4 compared to Q3 of IPWC. Adjusting for the IPI did not yield any significant changes in the estimates.

Further separation of Q5 into weight loss and gain (Figure S5) showed an elevated but imprecise risk of all‐cause mortality among those with weight gain (aHR [95% CI]: 1.17 [0.95–1.43]). Among those with normal pre‐pregnancy BMI, weight loss of less than 5 kg during the IPI (i.e., Q5 of IPWC) was associated with a higher risk of all‐cause mortality (aHR [95% CI]: 1.21 [1.02–1.43]) (Table S6). Additionally, those with weight retention or gain during the IPI (i.e., IPWC ≥ 0 kg) had an elevated risk of all‐cause mortality (aHR [95% CI]: 1.35 [1.01–1.79]). Adjustment for birth weight, gestational age at delivery, and pregnancy outcome (Table S7) yielded similar results.

The cause‐specific mortality risks across IPWC quintiles appeared comparable; however, these results were unstable and imprecise due to the small number of events, precluding meaningful interpretation (Table S8).

## 4 Discussion

In a large U.S. study with more than 50 years of follow‐up after pregnancy, maternal PPWR independently was associated with long‐term mortality risk, beyond the risk attributable to pre‐pregnancy BMI and GWG. Specifically, we found that women who returned to their pre‐pregnancy weight or maintained a weight slightly below that level had a lower risk of all‐cause mortality and experienced 55% lower diabetes‐related mortality compared to those with moderate PPWR between pregnancies. Furthermore, women who entered a subsequent pregnancy at a higher weight than their pre‐pregnancy baseline tended to have higher all‐cause mortality, although estimates were not statistically significant. Our analysis of IPWC, independent of GWG, suggested that participants who maintained or modestly reduced weight in the postpartum period had lower observed mortality, whereas those with weight retention or gain had higher observed mortality.

Our findings are novel and contribute to the growing body of literature on PPWR which highlights that women with high levels of PPWR face an increased risk of chronic conditions [12, 22, 23, 24]. For instance, Kew et al. [22] reported increased markers of adverse cardiometabolic health as early as 12 months after delivery, evidenced by higher mean adjusted diastolic blood pressure, insulin resistance, LDL cholesterol, and apolipoprotein B, along with declining adiponectin levels with higher weight gain trajectories after pregnancy [22]. Studies with longer follow‐up periods have reached similar conclusions regarding the long‐term health risk associated with PPWR. For example, Soria‐Contreras et al. [12] found that increasing postpartum weight trajectories were associated with higher levels of central adiposity, inflammation, and LDL cholesterol levels in later postpartum years [12]. Similarly, Kramer et al. [23] reported that PPWR was associated not only with a higher risk of prediabetes/diabetes at 5 years after delivery, but also predicted worse characterization of cardiovascular risk indices such as triglycerides, high‐/low‐density lipoprotein, apolipoprotein B, Matsuda index, insulin resistance, fasting glucose, and C‐reactive protein [23]. A study using data from the Danish National Birth Cohort (DNBC; 1997–2002) found elevated risk of hypertension and cardiovascular disease 16 years after delivery among women in the weight retention or gain groups [24]. Due to the small sample size, which may have contributed to the lack of precision in our cause‐specific results, we could not draw definitive conclusions about the increased risk of mortality from cardiovascular and kidney conditions associated with ICWC or IPWC within this cohort. However, our finding of a 55% reduction in diabetes‐related mortality from postpartum weight loss is notable. These results suggested that weight retention or gain during the postpartum period was associated with higher observed mortality, whereas weight loss was associated with lower observed mortality. Thus, our study not only complements previous studies that have linked PPWR to morbidity but also represents a novel contribution—being the first to map the association between PPWR and long‐term mortality and to suggest a potential survival benefit from healthy postpartum weight loss.

Our findings also underscore the need to implement postpartum weight management strategies across the full range of pre‐pregnancy BMI categories. In 2021, the US Preventive Services Task Force found strong evidence [39, 40, 41] that behavioral interventions reduce the likelihood of excessive weight gain during pregnancy, recommending that health care providers offer behavioral counseling to pregnant women to promote healthy weight gain in line with the National Academy of Medicine (NAM) 2009 guidelines [42]. However, such guidance for maintaining a healthy weight during the postpartum period remains limited. The few trials that have explored this area have focused on postpartum weight management for women living with overweight or obesity given their elevated risk of long‐term health complications, as is evident in this systematic review [43] of 18 randomized controlled trials (2559 participants). However, BMI‐specific analyses from this study suggest that women with low risk at baseline, i.e., a normal pre‐pregnancy BMI, who had weight retention or gain during the postpartum period had higher observed mortality. Therefore, postpartum weight management interventions should also consider normal BMI groups and not just women with BMI > 24.9 kg/m^2^.

This study focused on pregnant women from the 1950s and 1960s, utilizing a historic cohort to examine the long‐term health implications of PPWR, with follow‐up extending over 50 years—an important and unique strength of this study given it is unfeasible to study long‐term mortality utilizing contemporary cohorts. Yet some characteristics, such as prevalence of smoking and underlying causes of mortality in CPP, which differ from current obstetric populations, may limit generalizability. Other characteristics of the CPP cohort that may limit the generalizability of our findings include the underrepresentation of Hispanic women (2%–3% in CPP compared to ~26% in the 2023 U.S. obstetric population) [44] and a mean age about 7 years younger than the current mean age of childbearing persons (~30 years) [45]. Contemporary obstetric populations also exhibit a higher prevalence of GWG (50%) exceeding recommendations [46, 47, 48, 49, 50] compared with the CPP cohort (11.6%). While these differences may hinder direct generalization of our findings, CPP's rich covariate data and updated ICD‐coded mortality enable future application of transportability methods [51], which can “transport” results from the CPP‐era obstetric population (source population) to the current obstetric population (target population) despite differences in key characteristics—a valuable direction for future research. We were also unable to evaluate the effect of gestational diabetes mellitus or impaired glucose tolerance during pregnancy, screening for which was limited during the study period [30, 52]. Given the low prevalence of these conditions in our sample [30], there was limited statistical power to assess them as independent covariates or effect modifiers. In addition to any residual confounding, self‐reported pre‐pregnancy weight may also contribute to bias, but this is likely minimal given the high correlation with measured pregnancy‐related weight reported in other pregnancy cohorts [53, 54]. Despite these limitations, the study's analytical approach remains robust and is a key strength.

The analytical sample was restricted to women with at least two CPP‐registered pregnancies; however, the use of inverse probability weighting (IPW) accounted for potential selection bias introduced by this restriction, allowing the results to remain generalizable to the overall CPP population. The study's racial diversity, with nearly 50% of participants identifying as Black, is also a key strength, as much of the existing research on pregnancy exposures and long‐term outcomes has predominantly focused on White populations. Finally, while the IPWC analyses were adjusted for IPI, the strength of the association between IPWC and long‐term mortality may vary across different durations of IPI. Identifying such heterogeneity could help determine optimal time frames for postpartum weight management before a subsequent pregnancy. Future studies should explore potential effect modification by ICI, which was beyond the scope of this study due to sample size constraints. Similarly, while the IPWC analyses adjusted for GWG, the available sample size limits the ability to meaningfully evaluate GWG as an effect modifier, and this possibility should be explored in future studies.

## 5 Conclusion

In conclusion, returning to pre‐pregnancy weight or maintaining a weight slightly below that level was associated with alower long‐term mortality risk. This was true even among women with normal pre‐pregnancy BMI. These findings underscore the benefit of postpartum weight loss and suggest the need for effective strategies that support healthy weight maintenance post pregnancy. Future research with larger sample sizes and greater statistical power is needed to confirm these findings.

## Author Contributions

**Yajnaseni Chakraborti:** conceptualization, methodology, validation, formal analysis, writing – original/review and editing, visualization, project administration. **Sunni L. Mumford:** conceptualization, methodology, writing – review and editing. **Edwina H. Yeung:** conceptualization, methodology, data curation, writing – review and editing. **Katherine L. Grantz:** conceptualization, methodology, writing – review and editing. **Pauline Mendola:** conceptualization, methodology, writing – review and editing. **James L. Mills:** conceptualization, methodology, writing – review and editing. **Ellen C. Caniglia:** conceptualization, methodology, writing – review and editing. **Colleen M. Brensinger:** software, validation, formal analysis, writing – review and editing. **Cuilin Zhang:** conceptualization, methodology, writing – review and editing. **Enrique F. Schisterman:** conceptualization, methodology, writing – review and editing. **Stefanie N. Hinkle:** conceptualization, methodology, validation, investigation, resources, writing – review and editing, supervision.

## Funding

This research was supported, in part, by the Eunice Kennedy Shriver National Institute of Child Health and Human Development, National Institutes of Health (contract number HHSN275200800002I/27500013). Edwina H. Yeung and Katherine L. Grantz contributed to this work as part of their official duties as employees of the United States Federal Government.

## Conflicts of Interest

The authors declare no conflicts of interest.

## Supporting information


**Table S1:** Specification of analytical models in terms of CPP 1st pregnancy variables of interest.
**Figure S1:** Visual Assessment of Proportionality Assumption with respect to ICWC and IPWC quintiles (Imputed dataset 1).
**Figure S2:** Schoenfeld residual plots with respect to ICWC and IPWC quintiles (Imputed dataset 1).
**Figure S3:** Distribution of interconception weight change (ICWC) and interpregnancy weight change (IPWC).
**Figure S4:** Risk of all cause mortality associated with quintiles of interconception weight change (ICWC) where the top and bottom 1% were further separated out.
**Table S2:** All‐cause mortality risks associated with BMI‐specific quintiles of interconception weight change (ICWC) for those with Normal pre‐pregnancy BMI.
**Table S3:** Cause‐specific mortality associated with quintiles of interconception weight change (ICWC).
**Table S4:** Summary of participant characteristics across quintiles of interpregnancy weight change (ICWC).
**Table S5:** Distribution of outcome of interest across quintiles of interpregnancy weight change (IPWC).
**Figure S5:** Risk of all cause mortality associated with quintiles of interpregnancy weight change (IPWC) with weight gain group seperated out.
**Table S6:** All‐cause mortality risks associated with BMI‐specific quintiles of interpregnancy weight change (IPWC), and quintiles of IPWC with weight gain separated out for those with Normal pre‐pregnancy BMI.
**Table S7:** All‐cause mortality risks associated with quintiles of interpregnancy weight change (IPWC), and quintiles of IPWC with weight gain separated out adjusting for birth weight and gestational age at delivery.
**Table S8:** Cause‐specific mortality associated with quintiles of interpregnancy weight change (IPWC).

## Data Availability

Data from the Collaborative Perinatal Project are publicly available at https://www.archives.gov/research/electronic‐records/nih.html (National Archives Identifier: 606622). Researchers interested in the linked mortality data should contact the National Institute of Child Health and Human Development (Edwina H. Yeung, edwina.yeung@nih.gov) for details on a data‐sharing agreement and a confidentiality agreement with the National Death Index. The analysis codes are available from the corresponding author upon request.
