# Supplementary material for: Weight Change Between Pregnancies and Mortality Over 50 Years of Follow‐Up
Source: Obesity (Silver Spring). 2026 May 20;34(7):1502–15. doi: 10.1002/oby.70190 (PMC13306131; doi:10.1002/oby.70190)
Supplement: Supplementary file 1 — Table S1: Specification of analytical models in terms of CPP 1st pregnancy variables of interest. Figure S1: Visual Assessment of Proportionality Assumption with respect to ICWC and IPWC quintiles (Imputed dataset 1). Figure S2: Schoenfeld residual plots with respect to ICWC and IPWC quintiles (Imputed dataset 1). Figure S3: Distribution of interconception weight change (ICWC) and interpregnancy weight change (IPWC). Figure S4: Risk of all cause mortality associated with quintiles of interconception weight change (ICWC) where the top and bottom 1% were further separated out. Table S2: All‐cause mortality risks associated with BMI‐specific quintiles of interconception weight change (ICWC) for those with Normal pre‐pregnancy BMI. Table S3: Cause‐specific mortality associated with quintiles of interconception weight change (ICWC). Table S4: Summary of participant characteristics across quintiles of interpregnancy weight change (ICWC). Table S5: Distribution of outcome of interest across quintiles of interpregnancy weight change (IPWC). Figure S5: Risk of all cause mortality associated with quintiles of interpregnancy weight change (IPWC) with weight gain group seperated out. Table S6: All‐cause mortality risks associated with BMI‐specific quintiles of interpregnancy weight change (IPWC), and quintiles of IPWC with weight gain separated out for those with Normal pre‐pregnancy BMI. Table S7: All‐cause mortality risks associated with quintiles of interpregnancy weight change (IPWC), and quintiles of IPWC with weight gain separated out adjusting for birth weight and gestational age at delivery. Table S8: Cause‐specific mortality associated with quintiles of interpregnancy weight change (IPWC). [file OBY-34-1502-s001.pdf]

Appendix A: Data Cleaning and Processing

We preprocessed height and weight measurements by identifying and setting implausible values to missing based on predefined thresholds. Height discrepancies across pregnancies were resolved using the median value, while extreme values were flagged. Pre-pregnancy weight, delivery weight, and gestational weight gain (GWG) were set to missing if they fell outside biologically plausible ranges or deviated significantly (>±3 SD) from expected longitudinal trajectories. Weight measurements and rates of weight change between the two CPP pregnancies were further assessed in the context of both pregnancies, with extreme deviations flagged and set to missing. Using initial summary statistics of socio-demographic characteristics and other relevant information, we flagged and preprocessed height, pre-pregnancy weight, delivery weight, the 2nd pregnancy starting weight and derived weight measurements data by setting them to missing based on the following strategies. First, for all CPP participants, we examined height across pregnancies. Height was discrepant by ≥ 0.0762 m (3 in) between at least two of the repeated height measurements for 2.9% (n=200) of participants with at least two measurements. Thus, for all participants with more than one recorded height, the median between all height measurements was utilized. The minimum height overall was also cleaned to set any values < 0.1st percentile (1.2954 m; n=70) to missing; the 99.9th percentile for height was plausible (1.8288 m), as was the upper limit (2.032 m) and therefore was left as is. Second, for any pregnancies with pre-pregnancy BMI <15 (n=80; 0.2%) or >50 (n=29; 0.1%) kg/m^2 or pre-pregnancy weight <36.29 kg, the pre-pregnancy weight was set to missing. Weight at delivery and total gestational weight gain were also set to missing for any pregnancies with total gestational weight gain <-9.07 kg (-20 lbs.; n=123; 0.2%)) or < 36.29 kg (80 lbs.; n=20; <0.1%). Next, implausible pre-pregnancy weight (n=1118; 2.1%) and GWG (n=566; 1.1%) were assessed based on the context of their entire weight trajectory extracted from prenatal records using conditional percentiles[1]—implausible values were defined as those inconsistent with the expected weight distribution (>±3 SD) based on participants' longitudinal prenatal trajectory. These records were also set to be missing. Lastly, all weight measurements were cleaned based on the context of both pregnancies' weights and the time between the pregnancies. Weight measurements or their corresponding rate of weight change were set to missing for any records if either i) the difference or the rate of weight change between pre-pregnancy weights for 1st and 2nd pregnancies was < 0.1th (-16.8 kg; n=8; -0.041 kg/week; n=7) or > 99.9th (27.2 kg; n=9; 0.055 kg/week; n=8; <0.1%) percentile or ii) if the difference or rate of weight change between delivery weight for 1st pregnancy and weight at the start of the 2nd pregnancy was < 0.1th (-28.1 kg; n=9; -3.175 kg/week; n=8; <0.1%) or > 99.9th (14.1 kg; n=7; 0.558 kg/week; n=7; <0.1%).

1. Yang S, Hutcheon JA. Identifying outliers and implausible values in growth trajectory data. *Ann Epidemiol*. 2016;26(1):77-80.e2. doi:10.1016/j.annepidem.2015.10.002

Appendix B: Details on covariates used in different stages of the analysis in this study

Supplementary Table S1: Specification of analytical models in terms of CPP 1st pregnancy variables of interest

CPP 1st Pregnancy Variables of Interest	Definition and Functional Form in Analytical Models	Analytical Model		
		IPW [1]	ICWC [2]	IPWC [3]
Age (years)	Continuous	X	X	X
Race and Ethnicity	Categorical: White; Black; Puerto Rican; other †	X	X	X
Number of Prior Pregnancies	Categorical: 0; 1; 2; 3; 4	X	X	X
Marital Status	Categorical: single; married/common law; other ††.	X	X	X
Smoking Status	Categorical: Non-smoker; <1 pack/day; ≥ 1 pack/day	X	X	X
Annual Income	Categorical: ≤$1999; $2000-3999; $4000-5999; $6000-7999; $8000-9999; ≥$10000.	X	X	X
Education	Categorical: <High School; Some high school; High School Graduate; Some College.	X	X	X
Hypertensive disorders of pregnancy	Categorical: Normotensive; Chronic hypertension; Gestational hypertension; Preeclampsia/ Eclampsia; Superimposed Preeclampsia/Eclampsia	X		X
Prior Diabetes	Binary: Yes; No	X	X	X
Prior Cardiovascular Conditions	Binary: Yes; No	X	X	X
Prior Respiratory Conditions	Binary: Yes; No	X	X	X
Prior Renal Conditions	Binary: Yes; No	X	X	X
Prior Neurologic Conditions	Binary: Yes; No	X	X	X
Prior Cancer or Tumors	Binary: Yes; No	X	X	X
Pregnancy Plurality	Binary: Singleton; Multiples	X	X	X
Study Site	Categorical: Boston (reference); Buffalo; New Orleans; New York/Columbia; Baltimore; Virginia; Minnesota; New York/Medical College; Oregon; Pennsylvania; Providence; Tennessee	X	X	X
Pre-pregnancy BMI (kg/m^2) *	Continuous: calculated from self-reported pre-pregnancy weight and height.	X	X	X
Birth Weight (g)	Continuous: measured at delivery			X
Gestational Weight Gain (kg)	Continuous: calculated as the difference between weight measured at admission for delivery and self-reported pre-pregnancy weight			X
Gestational Age at Delivery (weeks)	Continuous: calculated based on date of delivery and date of LMP	X		X
Pregnancy outcome (denoting offspring(s) death/survival) ‡	Categorical: 0 = Live birth, stillbirth (reference); 1 = Pregnancy loss; 2 = Neonatal death; 3 = Infant or child death	X		X
Year of registration into CPP	Continuous	X		

CPP 1st Pregnancy Variables of Interest	Definition and Functional Form in Analytical Models	Analytical Model		
		IPW [1]	ICWC [2]	IPWC [3]
Interconception Interval (in days)	Continuous: calculated as the time difference between the LMP of the 1st and 2nd CPP pregnancies		X[a]	
Interpregnancy Interval (in years)	Continuous: calculated as the time difference between 1st pregnancy delivery date and LMP of the 2nd pregnancy			X[b]

[1] Inverse Probability Weighting Model; [2] Model with ICWC as exposure; [3] Model with IPWC as exposure

Ɨ Race and ethnicity were self-reported by participants during study enrollment through in-person interviews. The original CPP data collection forms categorized race as 'White,' 'Negro' (herein referred to as 'Black'), 'Oriental' (herein referred to as 'Asian'), 'Puerto Rican,' and 'Other.' In this manuscript, the 'Asian' and 'Other' categories were combined into a single 'Other' group due to the small number of participants who identified as Asian.

ƗƗ Other=widowed, divorced, separated

* BMI-stratified sensitivity analyses models exclude Pre-pregnancy BMI as a covariate. Instead, these models were only run among the Normal pre-pregnancy BMI group.

‡ Outcome codes were coded in the original CPP data collection forms as, 00 = Liveborn, still living; 01 = Abortion (≤19 weeks gestation); 02 = Abortion; 03 = Mole; 11 = Stillbirth (≥20 weeks gestation); 12 = Stillbirth (PRB Review); 29 = Fetal death, type unknown; 30 = Fetal death under 24 hours of age; 31 - 37 = Fetal death between day 1-7; 38 = Fetal death between 8–27 days; 39 = Neonatal death, time unknown; 40 = 28 days through 1 year; 51 = 1–2 years; 52 = 2 years 1 day to 3 years; 53 = 3 years 1 day to 4 years; 54 = 4 years 1 day to 5 years; 55 = 5 years 1 day to 6 years; 56 = 6 years 1 day to 7 years; 57 = 7 years 1 day to 8 years; 58 = Over 8 years; 59 = Child death, time unknown. In this manuscript, 00 = Liveborn, still living was coded as '0' and is the reference group; outcome codes 01, 02, 03, 11, 12, 29 were collapsed into the group '1' denoting the Pregnancy loss group; outcome codes 30, 31 – 37, 38, 39 were collapsed into the group '2' denoting the Neonatal death group; outcome codes 40, 51, 52, 53, 54, 55, 56, 57, 58, 59 were collapsed into the group '3' denoting the Infant or child death group.

[a] Included only for the ICWC model that was adjusted for ICI
[b] Included only for the IPWC model that was adjusted for IPI

Appendix C: Preliminary Analyses of Exposures and Outcomes

Supplementary Figure S1: Visual Assessment of Proportionality Assumption with respect to ICWC and IPWC Quintiles (Imputed dataset 1)

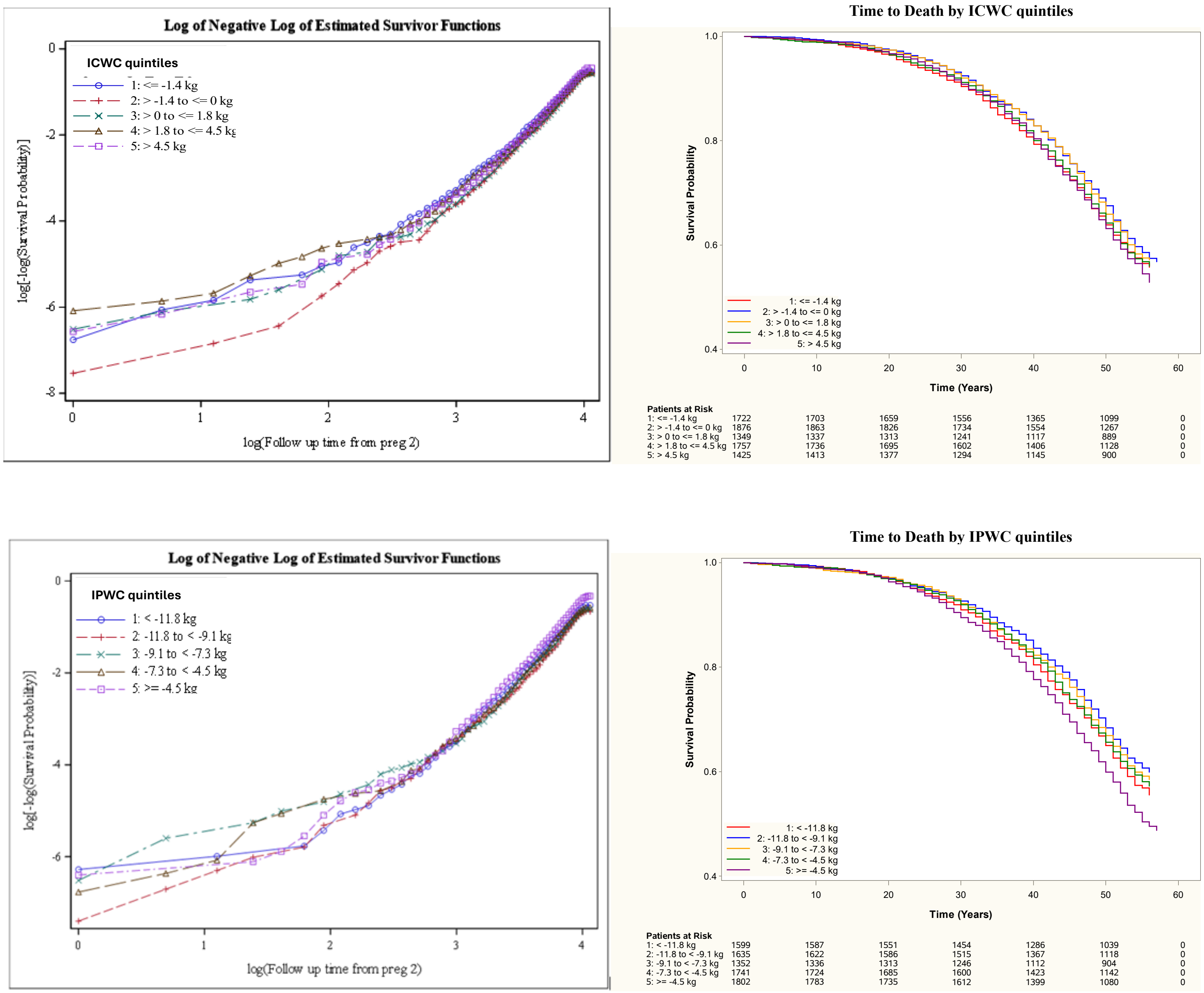

Supplementary Figure S2: Schoenfeld residual plots with respect to ICWC and IPWC Quintiles (Imputed dataset 1)

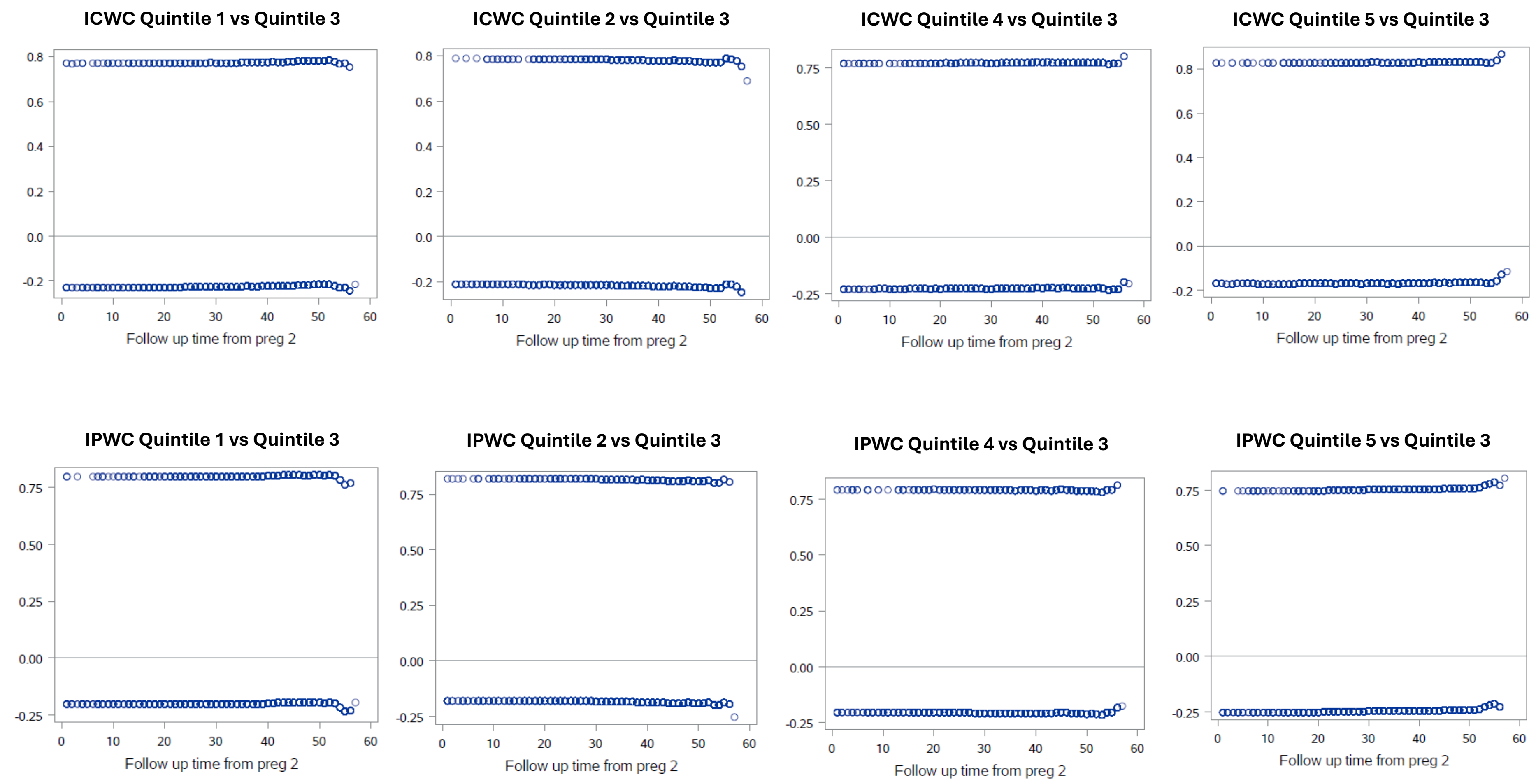

Supplementary Figure S3: Distribution of interconception weight change (ICWC) and interpregnancy weight change (IPWC)

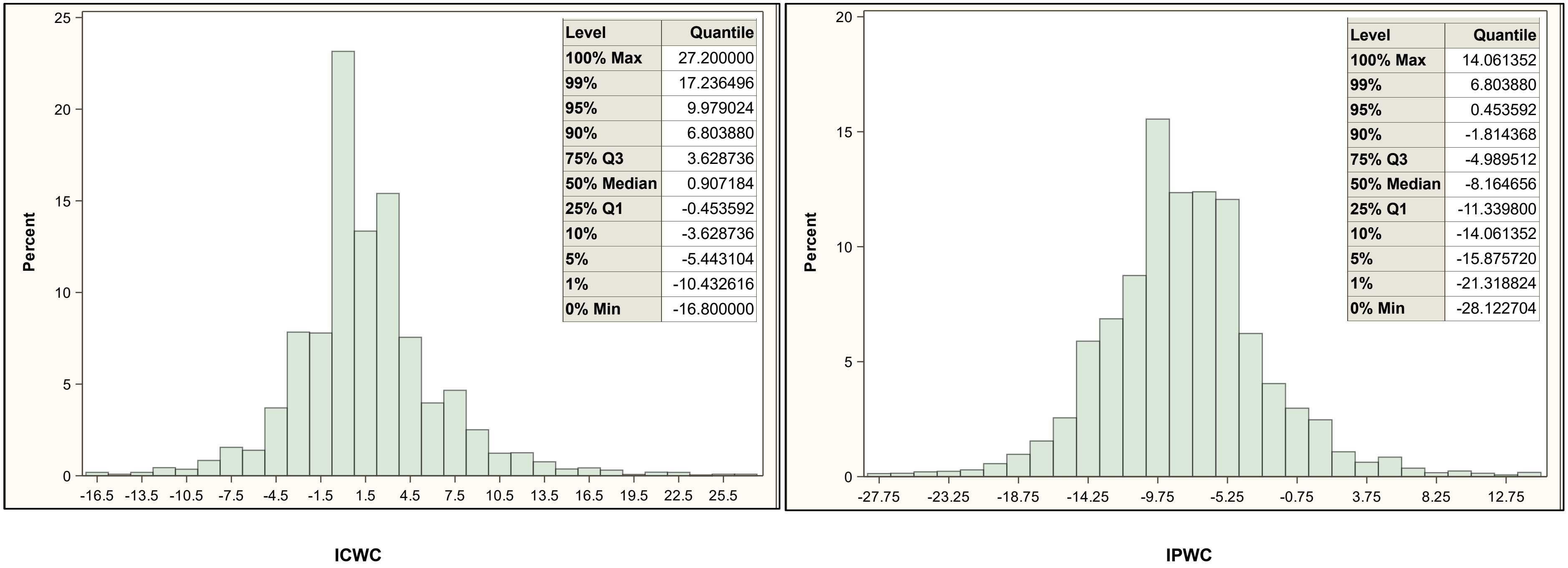

Appendix D: Findings from sensitivity and cause-specific mortality analyses associated with interconception weight change (ICWC)

Supplementary Figure S4: Risk of all cause mortality associated with quintiles of interconception weight change (ICWC) where the top and bottom 1% were further separated out

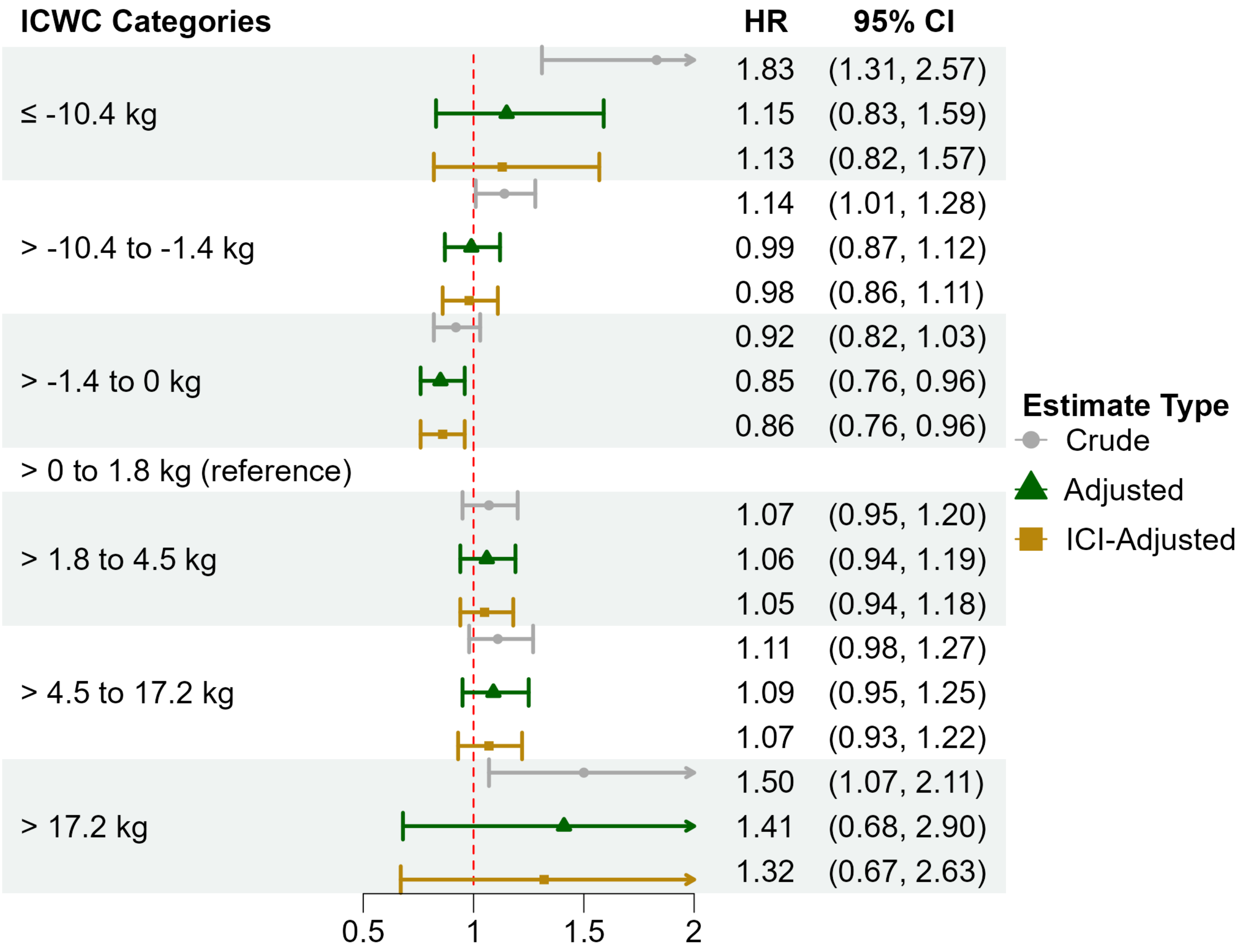

ICI: Interconception interval

Adjusted for CPP 1st Pregnancy variables: age, race and ethnicity, number of prior pregnancies, marital status, smoking status, annual income, education, prior diabetes, prior cardiovascular conditions, prior respiratory conditions, prior renal conditions, prior neurological conditions, prior cancer or tumors, pregnancy plurality, study site, and pre-pregnancy BMI

Supplementary Table S2: All-cause mortality risks associated with BMI-specific quintiles of interconception weight change (ICWC) for those with Normal pre-pregnancy BMI

Exposure	Category (in kg)	Unadjusted	Adjusted *	
		HR (95% CI)	HR (95% CI) adjusted without ICI	HR (95% CI) adjusted with ICI
BMI-specific quintiles of ICWC	Q1: ≤ -0.9	1.04 (0.90-1.20)	1.03 (0.87-1.22)	1.01 (0.85-1.20)
	Q2: > -0.9 to 0	0.87 (0.76-1.01)	0.85 (0.73-0.99)	0.86 (0.74-1.00)
	Q3: > 0 to 1.8	reference		
	Q4: > 1.8 to 4.5	1.07 (0.94-1.23)	1.08 (0.94-1.25)	1.07 (0.93-1.23)
	Q5: > 4.5	1.13 (0.95-1.33)	1.17 (0.99-1.40)	1.14 (0.96-1.36)

* Adjusted for CPP 1st Pregnancy variables: age, race and ethnicity, number of prior pregnancies, marital status, smoking status, annual income, education, prior diabetes, prior cardiovascular conditions, prior respiratory conditions, prior renal conditions, prior neurological conditions, prior cancer or tumors, pregnancy plurality, and study site

Supplementary Table S3: Cause-specific mortality associated with quintiles of interconception weight change (ICWC)

Cause of Death	Exposure (quintiles of ICWC in kg)	Unadjusted	Adjusted *	
		HR (95% CI)	HR (95% CI) adjusted without ICI	HR (95% CI) adjusted with ICI
**Cardiovascular**	Q1: ≤ -1.4	1.23 (0.98-1.54)	1.00 (0.79-1.27)	1.00 (0.79-1.26)
	Q2: > -1.4 to 0	0.98 (0.77-1.24)	0.88 (0.69-1.13)	0.89 (0.69-1.14)
	Q3: > 0 to 1.8	reference		
	Q4: > 1.8 to 4.5	1.08 (0.86-1.36)	1.03 (0.81-1.29)	1.02 (0.81-1.29)
	Q5: > 4.5	1.12 (0.86-1.46)	1.04 (0.80-1.34)	1.01 (0.79-1.30)
**Diabetes**	Q1: ≤ -1.4	1.22 (0.71-2.10)	0.76 (0.43-1.34)	0.75 (0.42-1.33)
	Q2: > -1.4 to 0	0.59 (0.33-1.06)	0.45 (0.23-0.87)	0.45 (0.24-0.87)
	Q3: > 0 to 1.8	reference		
	Q4: > 1.8 to 4.5	1.17 (0.67-2.04)	0.94 (0.51-1.74)	0.93 (0.51-1.72)
	Q5: > 4.5	1.80 (1.06-3.09)	1.42 (0.82-2.47)	1.39 (0.79-2.43)
**Kidney**	Q1: ≤ -1.4	0.58 (0.17-2.02)	0.41 (0.13-1.32)	0.41 (0.13-1.31)
	Q2: > -1.4 to 0	0.76 (0.28-2.04)	0.68 (0.26-1.81)	0.68 (0.26-1.81)
	Q3: > 0 to 1.8	reference		
	Q4: > 1.8 to 4.5	0.94 (0.41-2.17)	1.00 (0.41-2.45)	0.99 (0.41-2.42)
	Q5: > 4.5	1.47 (0.67-3.26)	1.20 (0.51-2.80)	1.18 (0.51-2.75)

* Adjusted for CPP 1st Pregnancy variables: age, race and ethnicity, number of prior pregnancies, marital status, smoking status, annual income, education, prior diabetes, prior cardiovascular conditions, prior respiratory conditions, prior renal conditions, prior neurological conditions, prior cancer or tumors, pregnancy plurality, study site, and pre-pregnancy BMI

Appendix E: Findings related to the secondary exposure variable

Supplementary Table S4: Summary of participant characteristics across quintiles of interpregnancy weight change

Participant Characteristics at 1st CPP Pregnancy	IPWC quintile					
	Q1: < -11.8 kg (n = 1329)	Q2: ≥-11.8 to <-9.1 kg (n = 1436)	Q3: ≥-9.1 to <-7.3 kg (n = 1218)	Q4: ≥-7.3 to <-4.5 kg (n = 1553)	Q5: ≥-4.5 kg (n = 1455)	Missing (n = 1174)
	n (%)	n (%)	n (%)	n (%)	n (%)	n (%)
Age (years)						
Mean (SD)	22.3 (5.0)	22.3 (5.1)	22.9 (5.2)	23.0 (5.5)	23.6 (5.6)	23.7 (5.7)
Missing	-	-	-	-	-	-
Race and Ethnicity						
Black	598 (45.0)	608 (42.3)	513 (42.1)	697 (44.9)	757 (52.0)	559 (47.6)
Asian/Other	15 (1.1)	14 (1.0)	15 (1.2)	8 (0.5)	6 (0.4)	11 (0.9)
Puerto Rican	37 (2.8)	37 (2.6)	46 (3.8)	43 (2.8)	52 (3.6)	77 (6.6)
White	679 (51.1)	777 (54.1)	644 (52.9)	805 (51.8)	640 (44.0)	527 (44.9)
Missing	-	-	-	-	-	-
Number of Prior Pregnancies						
0	553 (41.6)	633 (44.1)	514 (42.2)	586 (37.7)	482 (33.1)	358 (30.5)
1	256 (19.3)	291 (20.3)	232 (19.0)	323 (20.8)	289 (19.9)	208 (17.7)
2	155 (11.7)	179 (12.5)	168 (13.8)	225 (14.5)	209 (14.4)	156 (13.3)
3	131 (9.9)	119 (8.3)	105 (8.6)	140 (9.0)	154 (10.6)	144 (12.3)
4	230 (17.3)	210 (14.6)	194 (15.9)	274 (17.6)	298 (20.5)	266 (22.7)
Missing	*4 (0.3)*	*4 (0.3)*	*5 (0.4)*	*5 (0.3)*	*23 (1.6)*	*42 (3.6)*
Marital Status						
Married/Common Law	1018 (76.6)	1129 (78.6)	1006 (82.6)	1236 (79.6)	1159 (79.7)	942 (80.2)
Single	221 (16.6)	226 (15.7)	160 (13.1)	236 (15.2)	212 (14.6)	167 (14.2)
Widowed/Divorced/Separated	90 (6.8)	81 (5.6)	52 (4.3)	81 (5.2)	84 (5.8)	65 (5.5)
Missing	-	-	-	-	-	-
Smoking Status						
Nonsmoker	631 (47.5)	728 (50.7)	653 (53.6)	803 (51.7)	811 (55.7)	557 (47.4)
<1 pack per day	521 (39.2)	518 (36.1)	386 (31.7)	519 (33.4)	433 (29.8)	384 (32.7)
>= 1 pack per day	172 (12.9)	185 (12.9)	169 (13.9)	224 (14.4)	182 (12.5)	170 (14.5)
Missing	*5 (0.4)*	*5 (0.3)*	*10 (0.8)*	*7 (0.5)*	*29 (2.0)*	*63 (5.4)*
Annual Income						
<= $1999	201 (15.1)	215 (15.0)	141 (11.6)	209 (13.5)	225 (15.5)	165 (14.1)
$2000 - $3999	544 (40.9)	545 (38.0)	463 (38.0)	570 (36.7)	550 (37.8)	431 (36.7)
$4000 - $5999	252 (19.0)	290 (20.2)	257 (21.1)	351 (22.6)	287 (19.7)	225 (19.2)
$6000 - $7999	110 (8.3)	144 (10.0)	137 (11.2)	137 (8.8)	142 (9.8)	84 (7.2)
$8000 - $9999	39 (2.9)	54 (3.8)	53 (4.4)	57 (3.7)	50 (3.4)	25 (2.1)
>= $10000	19 (1.4)	22 (1.5)	37 (3.0)	30 (1.9)	26 (1.8)	14 (1.2)
Missing	*164 (12.3)*	*166 (11.6)*	*130 (10.7)*	*199 (12.8)*	*175 (12.0)*	*230 (19.6)*

Participant Characteristics at 1st CPP Pregnancy	IPWC quintile					
	Q1: < -11.8 kg (n = 1329)	Q2: ≥-11.8 to <-9.1 kg (n = 1436)	Q3: ≥-9.1 to <-7.3 kg (n = 1218)	Q4: ≥-7.3 to <-4.5 kg (n = 1553)	Q5: ≥-4.5 kg (n = 1455)	Missing (n = 1174)
	n (%)	n (%)	n (%)	n (%)	n (%)	n (%)
Education						
< High School	201 (15.1)	198 (13.8)	172 (14.1)	230 (14.8)	246 (16.9)	228 (19.4)
Some High School	592 (44.5)	591 (41.2)	470 (38.6)	623 (40.1)	573 (39.4)	450 (38.3)
High School Grad	387 (29.1)	456 (31.8)	402 (33.0)	475 (30.6)	446 (30.7)	285 (24.3)
>= Some College	111 (8.4)	152 (10.6)	143 (11.7)	168 (10.8)	119 (8.2)	107 (9.1)
Missing	*38 (2.9)*	*39 (2.7)*	*31 (2.5)*	*57 (3.7)*	*71 (4.9)*	*104 (8.9)*
Hypertensive disorders of pregnancy						
Normotensive	1257 (94.6)	1353 (94.2)	1146 (94.1)	1467 (94.5)	1328 (91.3)	1083 (92.2)
Chronic hypertension	24 (1.8)	20 (1.4)	27 (2.2)	32 (2.1)	60 (4.1)	41 (3.5)
Gestational hypertension	23 (1.7)	34 (2.4)	28 (2.3)	28 (1.8)	37 (2.5)	15 (1.3)
Preeclampsia/Eclampsia	10 (0.8)	13 (0.9)	10 (0.8)	9 (0.6)	16 (1.1)	16 (1.4)
Superimposed Preeclampsia/Eclampsia	15 (1.1)	15 (1.0)	7 (0.6)	17 (1.1)	14 (1.0)	11 (0.9)
Missing	-	*1 (0.1)*	-	-	-	*8 (0.7)*
Prior Diabetes †						
No	1307 (98.3)	1424 (99.2)	1198 (98.4)	1527 (98.3)	1421 (97.7)	1123 (95.7)
Yes	22 (1.7)	12 (0.8)	20 (1.6)	26 (1.7)	34 (2.3)	44 (3.7)
Missing	-	-	-	-	-	*7 (0.6)*
Prior Cardiovascular Conditions ‡						
No	1183 (89.0)	1253 (87.3)	1087 (89.2)	1360 (87.6)	1233 (84.7)	1020 (86.9)
Yes	145 (10.9)	182 (12.7)	131 (10.8)	193 (12.4)	222 (15.3)	141 (12.0)
Missing	*1 (0.1)*	*1 (0.1)*	-	-	-	*13 (1.1)*
Prior Respiratory Conditions §						
No	1241 (93.4)	1309 (91.2)	1141 (93.7)	1445 (93.0)	1353 (93.0)	1071 (91.2)
Yes	87 (6.5)	126 (8.8)	77 (6.3)	108 (7.0)	102 (7.0)	90 (7.7)
Missing	*1 (0.1)*	*1 (0.1)*	-	-	-	*13 (1.1)*
Prior Renal Conditions ¶						
No	1269 (95.5)	1382 (96.2)	1176 (96.6)	1477 (95.1)	1407 (96.7)	1102 (93.9)
Yes	59 (4.4)	53 (3.7)	42 (3.4)	76 (4.9)	48 (3.3)	59 (5.0)
Missing	*1 (0.1)*	*1 (0.1)*	-	-	-	*13 (1.1)*
Prior Neurological Conditions ||						
No	1220 (91.8)	1330 (92.6)	1122 (92.1)	1445 (93.0)	1340 (92.1)	1061 (90.4)
Yes	108 (8.1)	105 (7.3)	96 (7.9)	108 (7.0)	115 (7.9)	100 (8.5)
Missing	*1 (0.1)*	*1 (0.1)*	-	-	-	*13 (1.1)*
Prior Cancer or Tumors **						
No	1287 (96.8)	1375 (95.8)	1182 (97.0)	1508 (97.1)	1404 (96.5)	1136 (96.8)
Yes	41 (3.1)	60 (4.2)	36 (3.0)	45 (2.9)	51 (3.5)	25 (2.1)

Participant Characteristics at 1st CPP Pregnancy	IPWC quintile					
	Q1: < -11.8 kg (n = 1329)	Q2: ≥-11.8 to <-9.1 kg (n = 1436)	Q3: ≥-9.1 to <-7.3 kg (n = 1218)	Q4: ≥-7.3 to <-4.5 kg (n = 1553)	Q5: ≥-4.5 kg (n = 1455)	Missing (n = 1174)
	n (%)	n (%)	n (%)	n (%)	n (%)	n (%)
Missing	*1 (0.1)*	*1 (0.1)*	-	-	-	*13 (1.1)*
Pregnancy Plurality						
Singleton	1314 (98.9)	1423 (99.1)	1214 (99.7)	1541 (99.2)	1443 (99.2)	1125 (95.8)
Multiples	13 (1.0)	11 (0.8)	4 (0.3)	12 (0.8)	11 (0.8)	11 (0.9)
Missing	*2 (0.2)*	*2 (0.1)*	*0 (0.0)*	*0 (0.0)*	*1 (0.1)*	*38 (3.2)*
Study Site						
Boston	303 (22.8)	418 (29.1)	429 (35.2)	522 (33.6)	461 (31.7)	340 (29.0)
Buffalo	60 (4.5)	74 (5.2)	61 (5.0)	66 (4.2)	50 (3.4)	30 (2.6)
New Orleans	22 (1.7)	24 (1.7)	18 (1.5)	32 (2.1)	40 (2.7)	5 (0.4)
New York/Columbia	17 (1.3)	12 (0.8)	8 (0.7)	14 (0.9)	10 (0.7)	9 (0.8)
Baltimore	78 (5.9)	97 (6.8)	77 (6.3)	131 (8.4)	120 (8.2)	88 (7.5)
Virginia	54 (4.1)	82 (5.7)	50 (4.1)	88 (5.7)	108 (7.4)	93 (7.9)
Minnesota	97 (7.3)	103 (7.2)	62 (5.1)	55 (3.5)	29 (2.0)	41 (3.5)
New York/Medical College	40 (3.0)	33 (2.3)	43 (3.5)	40 (2.6)	48 (3.3)	72 (6.1)
Oregon	159 (12.0)	108 (7.5)	66 (5.4)	74 (4.8)	80 (5.5)	60 (5.1)
Pennsylvania	356 (26.8)	318 (22.1)	274 (22.5)	340 (21.9)	334 (23.0)	345 (29.4)
Providence	118 (8.9)	128 (8.9)	96 (7.9)	134 (8.6)	106 (7.3)	77 (6.6)
Tennessee	25 (1.9)	39 (2.7)	34 (2.8)	57 (3.7)	69 (4.7)	14 (1.2)
Missing	-	-	-	-	-	-
Pregnancy outcome						
0: liveborn	1279 (96.2)	1388 (96.7)	1159 (95.2)	1459 (93.9)	1185 (81.4)	971 (82.7)
1: pregnancy loss	11 (0.8)	15 (1.0)	17 (1.4)	45 (2.9)	196 (13.5)	120 (10.2)
2: neonatal death	14 (1.1)	11 (0.8)	21 (1.7)	35 (2.3)	55 (3.8)	29 (2.5)
3: infant/child death	23 (1.7)	20 (1.4)	21 (1.7)	14 (0.9)	18 (1.2)	16 (1.4)
Missing	2 (0.2)	2 (0.1)	0 (0.0)	0 (0.0)	1 (0.1)	38 (3.2)
Height (cm)						
Mean (SD)	162.0 (6.1)	161.2 (6.0)	160.6 (6.3)	160.5 (6.3)	160.7 (6.5)	160.5 (6.8)
Missing	*15 (1.1)*	*12 (0.8)*	*15 (1.2)*	*15 (1.0)*	*11 (0.8)*	*72 (6.1)*
Pre-pregnancy Weight (kg)						
Mean (SD)	59.2 (10.8)	56.4 (8.8)	56.6 (9.0)	57.2 (10.0)	61.6 (12.9)	60.2 (12.6)
Missing	-	-	-	-	-	*131 (11.2)*
Pre-pregnancy BMI (kg/m2)						
Mean (SD)	22.5 (3.9)	21.7 (3.1)	21.9 (3.2)	22.2 (3.6)	23.8 (4.7)	23.4 (4.6)
Median (IQR)	21.9 (19.9-24.2)	21.1 (19.6-23.2)	21.3 (19.8-23.2)	21.5 (19.7-23.9)	22.8 (20.5-26.1)	22.3 (20.3-25.0)
Missing	*15 (1.1)*	*12 (0.8)*	*15 (1.2)*	*15 (1.0)*	*11 (0.8)*	*173 (14.7)*
Pre-pregnancy BMI (kg/m2) categories						
Underweight: < 18.5 kg/m2	126 (9.5)	162 (11.3)	105 (8.6)	154 (9.9)	95 (6.5)	88 (7.5)

**Participant Characteristics at 1st CPP Pregnancy**	**IPWC quintile**					
	**Q1: < -11.8 kg (n = 1329)**	**Q2: ≥-11.8 to <-9.1 kg (n = 1436)**	**Q3: ≥-9.1 to <-7.3 kg (n = 1218)**	**Q4: ≥-7.3 to <-4.5 kg (n = 1553)**	**Q5: ≥-4.5 kg (n = 1455)**	**Missing (n = 1174)**
	**n (%)**	**n (%)**	**n (%)**	**n (%)**	**n (%)**	**n (%)**
Normal Weight: 18.5 – 24.9 kg/m2	923 (69.5)	1087 (75.7)	919 (75.5)	1118 (72.0)	876 (60.2)	656 (55.9)
Overweight: 25.0 – 29.9 kg/m2	197 (14.8)	146 (10.2)	145 (11.9)	210 (13.5)	317 (21.8)	163 (13.9)
Obese: > 30 kg/m2	68 (5.1)	29 (2.0)	34 (2.8)	56 (3.6)	156 (10.7)	94 (8.0)
Missing	*15 (1.1)*	*12 (0.8)*	*15 (1.2)*	*15 (1.0)*	*11 (0.8)*	*173 (14.7)*
Birth Weight (g)						
Mean (SD)	3373 (545.3)	3206 (517.3)	3125 (593.4)	3060 (640.9)	2886 (886.2)	3050 (803.0)
Missing	*6 (0.5)*	*5 (0.3)*	*5 (0.4)*	*12 (0.8)*	*111 (7.6)*	*121 (10.3)*
Gestational Weight Gain (kg)						
Mean (SD)	13.8 (4.5)	10.9 (3.3)	9.4 (3.4)	8.3 (3.7)	6.3 (4.8)	9.2 (4.8)
Missing	-	-	-	-	-	*603 (51.4)*
Gestational Age at Delivery (weeks)						
Mean (SD)	40.0 (3.0)	39.6 (3.2)	39.2 (3.3)	38.9 (3.9)	36.0 (8.0)	38.0 (6.1)
Missing	*7 (0.5)*	*5 (0.3)*	*2 (0.2)*	*4 (0.3)*	*7 (0.5)*	*17 (1.4)*
Interpregnancy Interval (in years) *						
Mean (SD)	1.2 (1.0)	1.1 (0.9)	1.1 (0.9)	1.2 (1.0)	1.4 (1.1)	1.3 (1.1)
Missing	*2 (0.2)*	*1 (0.1)*	-	*1 (0.1)*	*1 (0.1)*	*668 (56.9)*

Ɨ Type unknown. ‡ Cardiovascular conditions included hypertension, rheumatic fever, and any other cardiovascular diseases. §Respiratory conditions included tuberculosis, asthma, other chronic pulmonary diseases, and other conditions requiring thoracic surgery. ¶ Renal conditions included pyelitis, glomerulonephritis, and other conditions requiring kidney, urinary, or bladder surgery. ||Neurological conditions included neuromuscular diseases, convulsive disorders psychosis, alcohol or drug addiction, or other neurological diseases. **Cancer and tumors included any history of cancer, and gastrointestinal, kidney, urinary, bladder, or gynecological tumors.

* Interpregnancy Interval calculated as the time difference between 1st pregnancy delivery date and LMP of the 2nd pregnancy

Supplementary Table S5: Distribution of outcome of interest across quintiles of interpregnancy weight change (IPWC)

Outcomes of Interest		IPWC quintile					
		Q1: < -11.8 kg (n = 1329)	Q2: ≥-11.8 to <-9.1 kg (n = 1436)	Q3: ≥-9.1 to <-7.3 kg (n = 1218)	Q4: ≥-7.3 to <-4.5 kg (n = 1553)	Q5: ≥-4.5 kg (n = 1455)	Missing (n = 1174)
		n (%)	n (%)	n (%)	n (%)	n (%)	n (%)
Vital Status	Alive	771 (58.0)	900 (62.7)	737 (60.5)	926 (59.6)	767 (52.7)	659 (56.1)
	Dead	558 (42.0)	536 (37.3)	481 (39.5)	627 (40.4)	688 (47.3)	515 (43.9)
	Missing	*0 (0.0)*	*0 (0.0)*	*0 (0.0)*	*0 (0.0)*	*0 (0.0)*	*0 (0.0)*
Cause of Death	Cardiovascular	158 (28.3)	124 (23.1)	105 (21.8)	166 (26.5)	199 (28.9)	147 (28.5)
	Diabetes	27 (4.8)	19 (3.5)	14 (2.9)	19 (3.0)	25 (3.6)	32 (6.2)
	Kidney	10 (1.8)	2 (0.4)	11 (2.3)	7 (1.1)	20 (2.9)	9 (1.7)
	Missing	*47 (3.5)*	*63 (4.4)*	*52 (4.3)*	*59 (3.8)*	*51 (3.5)*	*53 (4.5)*

Supplementary Figure S5: Risk of all cause mortality associated with quintiles of interpregnancy weight change (IPWC) with weight gain group seperated out

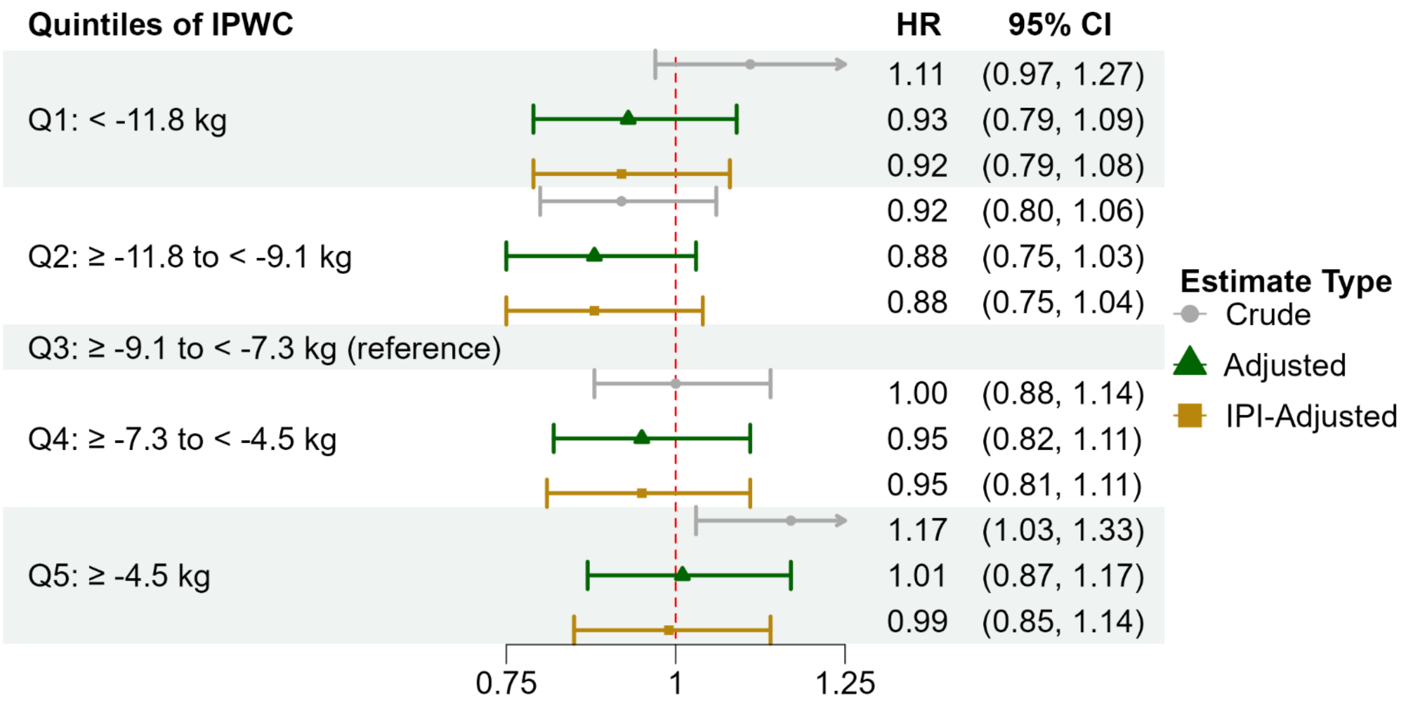

IPI: Interpregnancy interval

Adjusted for CPP 1st Pregnancy variables: age, race and ethnicity, number of prior pregnancies, marital status, smoking status, annual income, education, hypertensive disorders of pregnancy, prior diabetes, prior cardiovascular conditions, prior respiratory conditions, prior renal conditions, prior neurological conditions, prior cancer or tumors, pregnancy plurality, study site, pre-pregnancy BMI, and gestational weight gain

Supplementary Table S6: All-cause mortality risks associated with BMI-specific quintiles of interpregnancy weight change (IPWC), and quintiles of IPWC with weight gain separated out for those with Normal pre-pregnancy BMI

**Exposure**	**Category (in kg)**	**Unadjusted**	**Adjusted ***	
		**HR (95% CI)**	**HR (95% CI) adjusted without IPI**	**HR (95% CI) adjusted with IPI**
**BMI-specific quintiles of IPWC**	Q1: ≤ -11.8	1.18 (1.03-1.35)	0.98 (0.84-1.14)	0.97 (0.83-1.13)
	Q2: > -11.8 to -9.5	1.02 (0.88-1.19)	0.96 (0.82-1.14)	0.97 (0.82-1.14)
	Q3: > -9.5 to -7.3	reference		
	Q4: > -7.3 to -5.0	1.15 (0.99-1.34)	1.13 (0.96-1.33)	1.12 (0.95-1.31)
	Q5: > -5.0	1.17 (1.02-1.35)	1.21 (1.02-1.43)	1.17 (0.99-1.39)
**BMI-specific quintiles of IPWC with weight gain separated out**	1: ≤ -11.8	1.18 (1.03-1.35)	0.98 (0.84-1.14)	0.97 (0.83-1.13)
	2: > -11.8 to -9.5	1.02 (0.88-1.19)	0.96 (0.81-1.14)	0.97 (0.82-1.14)
	3: > -9.5 to -7.3	reference		
	4: > -7.3 to -5.0	1.15 (0.99-1.34)	1.13 (0.96-1.33)	1.12 (0.95-1.31)
	5: > -5.0 to < 0	1.13 (0.96-1.32)	1.17 (0.98-1.38)	1.14 (0.96-1.35)
	6: ≥ 0	1.34 (1.06-1.69)	1.35 (1.01-1.79)	1.29 (0.98-1.71)

* Adjusted for CPP 1st Pregnancy variables: age, race and ethnicity, number of prior pregnancies, marital status, smoking status, annual income, education, hypertensive disorders of pregnancy, prior diabetes, prior cardiovascular conditions, prior respiratory conditions, prior renal conditions, prior neurological conditions, prior cancer or tumors, pregnancy plurality, study site, and gestational weight gain

Supplementary Table S7: All-cause mortality risks associated with quintiles of interpregnancy weight change (IPWC), and quintiles of IPWC with weight gain separated out adjusting for birth weight and gestational age at delivery

Exposure	Category (in kg)	Birth weight-Adjusted *		Gestational age at delivery-Adjusted *		Pregnancy Outcome-Adjusted *	
		HR (95% CI) adjusted without IPI	HR (95% CI) adjusted with IPI	HR (95% CI) adjusted without IPI	HR (95% CI) adjusted with IPI	HR (95% CI) adjusted without IPI	HR (95% CI) adjusted with IPI
Quintiles of IPWC	Q1: < -11.8	0.93 (0.79-1.09)	0.92 (0.79-1.08)	0.93 (0.79-1.09)	0.92 (0.79-1.08)	0.93 (0.79-1.09)	0.92 (0.79-1.08)
	Q2: ≥ -11.8 to < -9.1	0.88 (0.75-1.03)	0.88 (0.75-1.04)	0.88 (0.75-1.03)	0.88 (0.75-1.04)	0.88 (0.75-1.03)	0.88 (0.75-1.04)
	Q3: > -9.1 to -7.3	reference					
	Q4: ≥ -7.3 to < -4.5	0.95 (0.82-1.11)	0.95 (0.81-1.10)	0.95 (0.82-1.11)	0.95 (0.81-1.11)	0.96 (0.82-1.11)	0.95 (0.81-1.11)
	Q5: ≥ -4.5	0.99 (0.86-1.15)	0.97 (0.84-1.13)	1.01 (0.87-1.17)	0.99 (0.85-1.15)	1.01 (0.87-1.17)	0.99 (0.85-1.14)
Quintiles of IPWC with weight gain separated out	1: < -11.8	0.92 (0.79-1.09)	0.92 (0.79-1.08)	0.93 (0.79-1.09)	0.92 (0.79-1.08)	0.93 (0.79-1.09)	0.92 (0.79-1.08)
	2: ≥ -11.8 to < -9.1	0.88 (0.75-1.03)	0.88 (0.75-1.04)	0.88 (0.75-1.03)	0.88 (0.75-1.04)	0.88 (0.75-1.03)	0.88 (0.75-1.04)
	3: > -9.1 to -7.3	reference					
	4: ≥ -7.3 to < -4.5	0.95 (0.82-1.11)	0.95 (0.81-1.11)	0.96 (0.82-1.12)	0.95 (0.81-1.11)	0.96 (0.82-1.12)	0.95 (0.82-1.11)
	5: ≥ -4.5	0.95 (0.81-1.10)	0.93 (0.80-1.08)	0.96 (0.82-1.12)	0.94 (0.81-1.10)	0.96 (0.82-1.12)	0.94 (0.81-1.10)
	6: ≥ 0	1.14 (0.93-1.40)	1.10 (0.90-1.35)	1.17 (0.95-1.44)	1.13 (0.92-1.39)	1.17 (0.95-1.43)	1.13 (0.92-1.38)

* Adjusted for CPP 1st Pregnancy variables: age, race and ethnicity, number of prior pregnancies, marital status, smoking status, annual income, education, hypertensive disorders of pregnancy, prior diabetes, prior cardiovascular conditions, prior respiratory conditions, prior renal conditions, prior neurological conditions, prior cancer or tumors, pregnancy plurality, study site, pre-pregnancy BMI, and gestational weight gain

Supplementary Table S8: Cause-specific mortality associated with quintiles of interpregnancy weight change (IPWC)

Cause of Death	Exposure (quintiles of IPWC in kg)	Unadjusted	Adjusted *	
		HR (95% CI)	HR (95% CI) adjusted without IPI	HR (95% CI) adjusted with IPI
**Cardiovascular**	Q1: < -11.8	1.25 (0.88-1.77)	1.02 (0.69-1.51)	1.01 (0.69-1.49)
	Q2: ≥ -11.8 to < -9.1	0.99 (0.72-1.35)	0.98 (0.69-1.38)	0.98 (0.69-1.38)
	Q3: > -9.1 to -7.3	reference		
	Q4: ≥ -7.3 to < -4.5	1.19 (0.88-1.60)	1.15 (0.83-1.60)	1.14 (0.83-1.58)
	Q5: ≥ -4.5	1.38 (0.98-1.94)	1.11 (0.78-1.57)	1.08 (0.76-1.53)
**Diabetes**	Q1: < -11.8	1.68 (0.95-2.95)	0.87 (0.45-1.70)	0.87 (0.45-1.69)
	Q2: ≥ -11.8 to < -9.1	0.83 (0.44-1.57)	0.85 (0.42-1.69)	0.86 (0.43-1.71)
	Q3: > -9.1 to -7.3	reference		
	Q4: ≥ -7.3 to < -4.5	1.07 (0.58-1.98)	1.13 (0.60-2.14)	1.11 (0.59-2.11)
	Q5: ≥ -4.5	1.26 (0.75-2.13)	1.05 (0.59-1.86)	1.01 (0.56-1.81)
**Kidney**	Q1: < -11.8	1.05 (0.42-2.64)	0.72 (0.26-2.01)	0.72 (0.26-1.99)
	Q2: ≥ -11.8 to < -9.1	0.42 (0.09-1.95)	0.37 (0.08-1.75)	0.38 (0.08-1.76)
	Q3: > -9.1 to -7.3	reference		
	Q4: ≥ -7.3 to < -4.5	0.80 (0.25-2.51)	0.80 (0.24-2.65)	0.80 (0.24-2.61)
	Q5: ≥ -4.5	2.12 (0.89-5.04)	1.75 (0.68-4.47)	1.72 (0.68-4.37)

* Adjusted for CPP 1st Pregnancy variables: age, race and ethnicity, number of prior pregnancies, marital status, smoking status, annual income, education, hypertensive disorders of pregnancy, prior diabetes, prior cardiovascular conditions, prior respiratory conditions, prior renal conditions, prior neurological conditions, prior cancer or tumors, pregnancy plurality, study site, pre-pregnancy BMI, and gestational weight gain
